# Fundamental principles of photoelectrochemical sensors with focus on hexavalent chromium detection

**DOI:** 10.1039/d5ra06395g

**Published:** 2025-10-24

**Authors:** Yaroslav Zhigalenok, Aigerim Tazhibayeva, Saule Kokhmetova, Alena Starodubtseva, Tatyana Kan, Fyodor Malchik

**Affiliations:** a Al-Farabi Kazakh National University Almaty 050040 Kazakhstan tornatore@mail.ru

## Abstract

Hexavalent chromium constitutes a major hazard to the environment and human health. Consequently, monitoring methods capable of widespread deployment are necessary. However, traditional laboratory analytical methods, despite their accuracy, prove unsuitable for mass screening due to high cost and the need for stationary equipment. Photoelectrochemical sensors provide a promising alternative as they allow portable, cost-effective devices with low detection limits and inherent selectivity to chromium oxidation states. This review analyzes the current state of photoelectrochemical detection of Cr(vi), from fundamental principles to practical applications. The physicochemical foundations of PEC sensor operation are examined in detail: semiconductor–light interactions, electrolyte interface formation, and charge carrier generation and separation mechanisms. Major materials science strategies for improving analytical characteristics are analyzed: creation of semiconductor heterostructures, use of quantum dots and plasmonic nanostructures, transport network engineering, and molecular surface modification. The review shows the development from simple single-phase semiconductor systems to complex multi-component architectures designed to overcome fundamental limitations. Progress in materials design has allowed notable improvements in sensor performance through rational engineering of optical, electronic, and surface properties. Analysis of Cr(vi) detection systems shows general design principles that can be applied to photoelectrochemical sensing of other environmental contaminants. We provide an overview for researchers in photocatalysis, electrochemistry, and materials chemistry working on next-generation photoactive analytical systems.

## Abstract 2

The review analyzes photoelectrochemical Cr(vi) sensing from semiconductor basics to material engineering. Strategies include heterostructures, quantum dots, plasmonic structures, conductive networks and molecular recognition.

## Graphical abstract

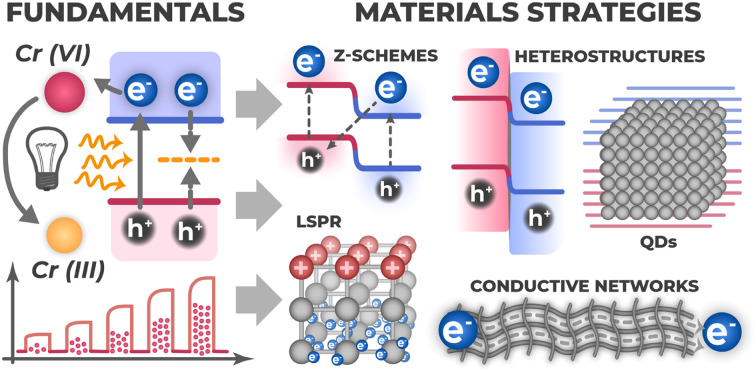

## 1. Introduction

Hexavalent chromium (Cr(vi)) is a highly toxic compound that has been used for decades in electroplating, leather tanning, pigment production, metallurgy, and wood preservation.^1–3^ Massive discharges of chromium-containing wastewater have made Cr(vi) one of the most widespread pollutants in industrial effluents, soils, and drinking water sources worldwide. The International Agency for Research on Cancer (IARC) classifies Cr(vi) as a group 1 carcinogen – a substance with proven ability to cause cancer in humans.^4^

The problem of chromium contamination is large-scale. Industrial and natural sources form extensive foci of soil and groundwater contamination that span vast territories and persist for decades.^5^ This situation necessitates mass and regular screening. However, traditional laboratory analytical methods – inductively coupled plasma mass spectrometry (ICP-MS), liquid chromatography, and atomic absorption spectroscopy (AAS) – despite their accuracy, prove ineffective for this task due to high analysis costs, the need for stationary equipment, and lengthy sample preparation.^6–8^

The search for alternative approaches has stimulated active development of various sensor technologies aimed at creating portable and rapid analytical methods, including colorimetric,^9,10^ fluorescent,^11,12^ and electrochemical approaches.^13,14^ Colorimetric sensors often provide only qualitative or semi-quantitative results with limited sensitivity, while fluorescence methods require specialized equipment and prolonged analysis times.^7^ Electrochemical sensors combine low cost and rapid response times with detection limits in the nanomolar range.^8^

Photoelectrochemical sensors inherit these electrochemical advantages while adding light as an external stimulus, which allows for signal generation with minimal background interference. They are based on direct conversion of light energy into electric current, which provides an exceptionally high signal-to-noise ratio due to low background current in darkness,^15,16^ allowing achievement of very low detection limits.^17^ The ability to operate under natural illumination creates prerequisites for developing field analytical devices. Additionally, PEC methods possess intrinsic selectivity to element oxidation states, which is critically important for distinguishing toxic Cr(vi) from relatively harmless Cr(iii).^18^

There are numerous review works demonstrating the breadth of PEC sensor applications. The main research focus is concentrated on several directions. Significant attention is devoted to biomedical diagnostics, where highly sensitive platforms are being developed for detection of cancer markers^19^ (carcinoembryonic antigen^20^), cardiovascular biomarkers,^21^ as well as nucleic acids^22^ and other biologically active molecules. Another crucial direction is environmental monitoring^23^ and food safety.^24^ Within this theme, sensors are being created for analysis of pesticides,^25^ antibiotics,^26^ mycotoxins,^27^ and industrial pollutants such as bisphenol A.^28^

Moreover, many reviews are devoted not so much to specific analytes as to the development and functionalization of photoactive materials themselves, which form the basis for next-generation sensors. These include, for example, bismuth oxyhalides,^29^ various two-dimensional nanomaterials,^30–32^ and porous crystalline structures such as metal–organic frameworks (MOFs) and covalent organic frameworks (COFs).^33^

From the perspective of chromium detection, both general analytical method reviews^34,35^ and reviews of specific approaches exist, such as electrochemical,^8,36^ spectrophotometric,^37,38^ and fluorescent methods. However, review works dedicated to hexavalent chromium detection using photoelectrochemical sensors are absent to our knowledge. Given the complex chemistry of chromium, the growing popularity of photoelectrochemical sensors, and the multidisciplinary nature of this topic, such a review is needed.

Furthermore, typically, most review works focus on describing and listing synthesis methods, morphology, and analytical characteristics of specific systems. The narrow focus of most publications leads to insufficient coverage of fundamental principles of sensor operation, which complicates understanding of their working principles for specialists from related fields.

In the present work, we begin with detailed exposition of fundamental physicochemical principles of PEC detection, and then use Cr(vi) sensors as illustrative examples to demonstrate specific materials science strategies. Emphasis is placed on analysis of mechanisms and operating principles followed by evaluation of the entire field. Therefore, this review will be useful both for PEC sensor specialists and for a broad range of researchers in photocatalysis, electrochemistry, and materials chemistry.

## 2. Fundamental principles of photoelectrochemical sensors

Photoelectrochemical sensors are multicomponent systems where light energy is converted into an analytically useful electrical signal through interconnected physicochemical processes. Their operation involves phenomena occurring at different scales – from quantum-mechanical processes of photon absorption and charge carrier generation to macroscopic electrochemical reactions at phase interfaces.

Photoelectrochemical sensor operation depends on the electronic properties of semiconductor materials, determined by their band structure. Semiconductors must efficiently absorb light in a certain spectral range, generate electron–hole pairs, and spatially separate them to create photoactive systems. To transform these primary photophysical processes into a stable and reproducible analytical signal, understanding phenomena occurring at the semiconductor–electrolyte interface becomes important.

The efficiency and selectivity of photoelectrochemical detection depend on electric double layer formation, energy band bending, interfacial charge transfer, and competition between analytical reactions and parasitic recombination.

### 2.1. Semiconductors and light–matter interactions

**Fig. 1:**
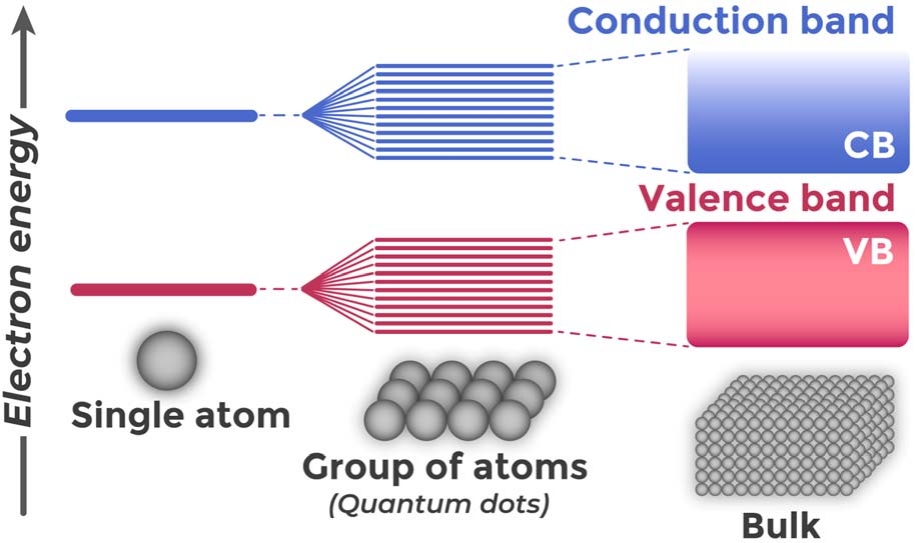
Schematic presentation of the formation of allowed energy bands (valence and conduction) in a solid during transition from discrete levels of an individual atom to bulk material.

Semiconductor properties derive from the band theory of solids.^39^ When isolated atoms combine into a crystal lattice, their electronic orbitals overlap and interact. This interaction follows quantum mechanical principles: when atomic spacing becomes comparable to electron cloud dimensions, wave functions from neighboring atoms overlap, causing orbital hybridization and creating bonding and antibonding molecular orbitals. With increasing numbers of interacting atoms, molecular orbitals split into numerous closely spaced energy states.^40^ Since a crystal contains an enormous number of atoms (for example, for silicon 5 × 10^22^ cm^−3^ (ref. 41)), these sublevels are positioned so close to each other in energy that they form continuous allowed energy bands,^42^ as illustrated in Fig. 1.

The periodic crystal lattice structure creates forbidden energy bands where no electronic states can exist.^43^ The uppermost allowed band that is completely filled with electrons at absolute zero temperature is called the valence band (VB). Directly above it is located the conduction band (CB) – the next allowed band that is empty at 0 K in semiconductors and dielectrics. The energy interval between the top of the VB and the bottom of the CB determines the band gap width (*E*_g_).

**Fig. 2:**
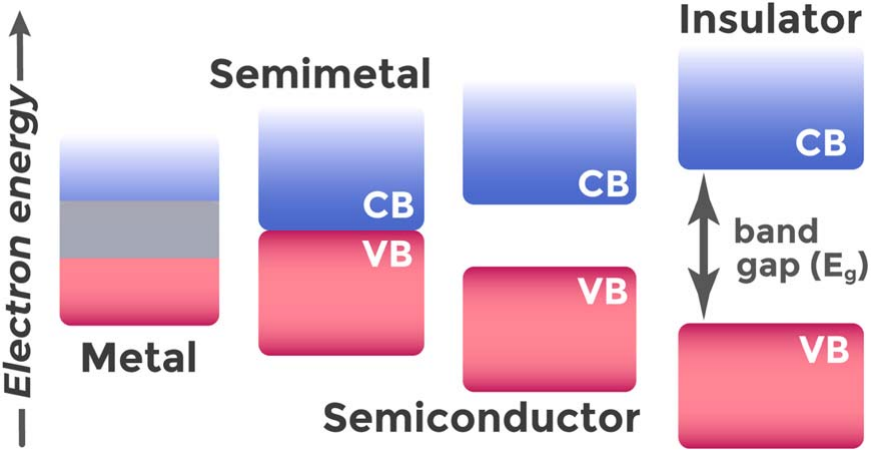
Classification of materials into metals, semiconductors, and dielectrics depending on band filling and band gap width (*E*_g_).

The nature of filling these bands and the band gap width allow classification of materials,^43^ as shown in Fig. 2. In metals, the valence band is either partially filled or overlaps with the conduction band, which results in high electron concentration and good electrical conductivity. Semimetals (such as graphite, bismuth, arsenic^43^) occupy an intermediate position between metals and semiconductors. Their valence and conduction bands slightly overlap or touch, providing small charge carrier concentrations and intermediate conductivity. In dielectrics, conversely, the valence band is completely filled, and the conduction band is separated by a very wide forbidden band, so significant energies are required to transfer electrons to the conduction band, and their electrical conductivity is extremely low.

**Fig. 3:**
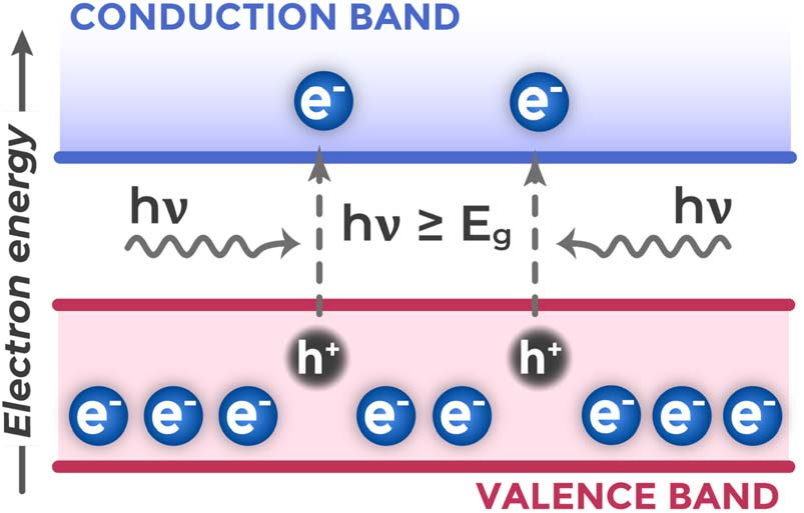
Principle of photogeneration of electron–hole pairs in a semiconductor.

Semiconductors also have completely filled valence bands and empty conduction bands at 0 K, but with relatively small band gaps (1–4 eV (ref. 39)). At room temperature or under external influences (light, electric fields, radiation), electrons can transition from valence to conduction band. The band gap *E*_g_ sets the minimum photon energy for this transition and determines which wavelengths the semiconductor can efficiently absorb. Thus, narrow-band-gap semiconductors (with small *E*_g_) are sensitive to lower-energy photons (visible and infrared light),^44,45^ while wide-band-gap semiconductors (with large *E*_g_) require more energetic photons for excitation (ultraviolet or blue light).^46^Fig. 3 illustrates the principle of electron–hole pair photogeneration in a semiconductor under illumination.

Electrons that have transitioned to the CB become free carriers of negative charge. Electrons in the VB are bound in covalent bonds between atoms and localized in specific regions of the crystal. Electrons in the CB have access to many unoccupied energy states, which enables them to change their momentum under external fields and participate in current transport. Simultaneously, in the VB, a vacant state forms at the location of the departed electron, which behaves as a positively charged quasiparticle – a hole, also capable of participating in charge transport through hopping of neighboring electrons to this vacant position.^43^

The Fermi level (*E*_F_) is an energy level whose probability of being occupied by an electron at thermodynamic equilibrium is exactly 1/2.^43^ In metals, the Fermi level is located within the partially filled conduction band and corresponds to the boundary between filled and empty electronic states. In semiconductors, the valence band is filled and the conduction band is mostly empty, so the Fermi level is located in the band gap – a region where electronic states are absent. The Fermi level position in the band gap reflects the statistical distribution of electrons between the VB and CB at a given temperature.

From a thermodynamic perspective, the Fermi level corresponds to the electrochemical potential of electrons (*μ*) – the energy required to add one electron to the system at constant temperature and volume.^43^ It is important to note that the term “electrochemical potential” in the context of solids includes both the chemical component (related to carrier concentration) and the electrical component (related to the internal electric field). The alignment of electrochemical potentials drives charge transfer upon contact of different materials.

In intrinsic (undoped) semiconductors, the concentration of electrons in the CB equals the concentration of holes in the VB, since each thermally excited electron leaves behind a hole. In this case, the Fermi level is located approximately in the middle of the band gap.

Semiconductor properties can be purposefully modified through doping – the introduction of small amounts of impurity atoms. Donor impurities are atoms that create additional energy levels within the band gap but very close to the bottom of the conduction band. For example, atoms of group V elements (P, As) in a silicon lattice (group IV) have one extra valence electron.^47^ In an isolated impurity atom, this electron would be tightly bound by Coulomb attraction to the nucleus. However, in a crystal, between the electron and nucleus is a medium with high dielectric permittivity (for silicon *ε* ≈ 11.7 (ref. 43)). This means that the nucleus's electric field is weakened by a factor of *ε*, which reduces the electron attraction force by the same factor. Additionally, the effective mass of the electron in the crystal is less than that of a free electron. As a result, the binding energy of the extra electron to the donor atom is on the order of tens of meV (for phosphorus impurity in silicon crystal, the ionization energy is 0.045 eV (ref. 43)), which is two orders of magnitude less than in an isolated atom (for phosphorus, the first ionization energy is 10.49 eV (ref. 48)). At room temperature (*k*_b_*T* ≈ 0.025 eV (ref. 49)), thermal energy is sufficient to detach the electron from the donor and transfer it to the conduction band. Each ionized donor becomes an immobile positive ion, maintaining crystal electroneutrality.

**Fig. 4:**
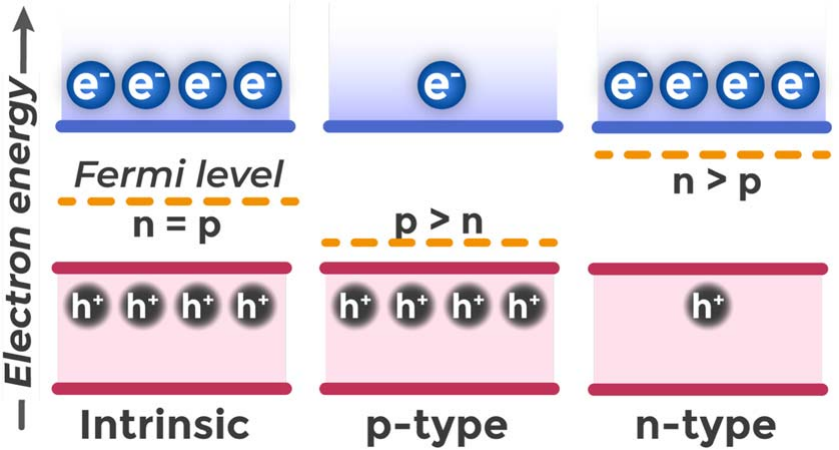
Position of the Fermi level (*E*_F_) and charge carrier concentrations (electrons n and holes p) in intrinsic (undoped), p-type, and n-type semiconductors.

Acceptor impurities (group III elements for silicon) have one fewer valence electron and create unfilled energy levels in the band gap near the top of the valence band. By capturing electrons from the valence band through thermal energy, they create holes – the majority charge carriers in p-type semiconductors. Accordingly, to ensure high hole concentration, the Fermi level must shift downward toward the top of the valence band so that the probability of electron absence (hole presence) in the valence band becomes significant Fig. 4 compares the Fermi level positions and majority carrier concentrations in intrinsic (undoped), p-type, and n-type semiconductors.

An important property of semiconductors is their ability to absorb light and generate free charge carriers. The basic condition for such interband photon absorption by a semiconductor is that the photon energy (*E* = *hν*, where *h* is Planck's constant, *ν* is light frequency^50^) exceeds the band gap width.^51^ If the photon energy is less than *E*_g_, it cannot cause a direct interband electron transition, and light in an ideal pure semiconductor mostly passes through it or reflects from the surface without creating electron–hole pairs. The energy of an individual photon is determined by the frequency of the light wave, not its amplitude. This follows from the quantum nature of light: each photon carries a fixed portion of energy, which depends only on the radiation frequency. Wave amplitude determines light intensity – the number of photons passing through a unit area per unit time – but does not affect the energy of each individual photon.

When the condition *hν* ≥ *E*_g_ is satisfied, the photon is absorbed, and its energy is spent on transferring an electron from the VB to the CB. A material's ability to absorb light is quantitatively characterized by the absorption coefficient (*α*), which determines how intensely light is attenuated when passing through a substance according to the Beer–Lambert law.^49^

**Fig. 5:**
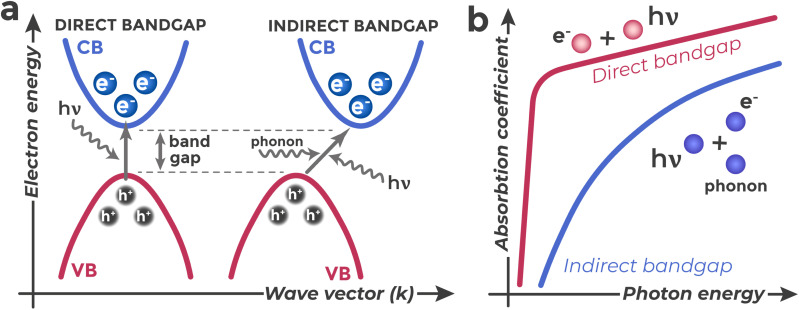
Schematic presentation of direct and indirect interband optical transitions (a) and their corresponding dependence of absorption coefficient (*α*) on photon energy (*hν*) (b).

The probability and nature of interband optical transitions substantially depend on details of the semiconductor's energy band structure, particularly on the mutual arrangement of extrema (VB maximum and CB minimum) in the space of electron wave vectors (quasimomentum or crystal momentum), *k*^52^ (Fig. 5a). The wave vector characterizes the quantum state of an electron in the periodic field of a crystal. Quasimomentum is a generalization of the ordinary momentum concept for electrons moving in the periodic potential of a crystal lattice. Unlike free electrons in vacuum, electrons in a crystal interact with regularly arranged atoms, leading to modification of their momentum characteristics.

In direct band gap semiconductors (*e.g.*, GaAs, InGaAsP^53^), the CB minimum and VB maximum are located at the same wave vector value (Δ*k* ≈ 0),^53^ as presented in Fig. 5a. In this case, electron transition can occur with absorption of only a photon, since photon momentum is negligibly small compared to characteristic changes in electron quasimomentum during interband transitions.^52^ Such direct transitions have high probability, resulting in sharp increase of the absorption coefficient near the fundamental absorption edge (the region of photon energies *hν* ≈ *E*_g_).

In indirect band gap semiconductors (*e.g.*, Si^53^), the CB minimum and VB maximum are displaced relative to each other in *k*-space (Δ*k* ≠ 0). For an interband transition to occur, the electron must change not only its energy (through photon absorption) but also its quasimomentum. This requirement follows from fundamental conservation laws that must be fulfilled in any physical processes in a crystal. Just as energy and momentum are simultaneously conserved in mechanics, energy and quasimomentum must be conserved in a crystal.^54^ Since a photon does not transfer momentum to an electron, to satisfy the momentum conservation law, a third particle must participate in such a transition – a phonon, which is a quantum of crystal lattice vibrations and possesses both energy and quasimomentum.^53^ The electron simultaneously interacts with a photon and phonon (absorbing or emitting it). Phonons are constantly present in a crystal due to thermal energy.^43^ Such a three-particle process (electron–photon–phonon) is less probable than a direct two-particle transition. Therefore, in indirect band gap semiconductors, light absorption precisely near the band edge (at *hν* ≈ *E*_g_) is characterized by a lower absorption coefficient and more gradual dependence on photon energy compared to direct band gap materials (Fig. 5b). At photon energies significantly exceeding *E*_g_, other, more efficient transitions may become possible^55^ and differences in absorption are smoothed out.

**Fig. 6:**
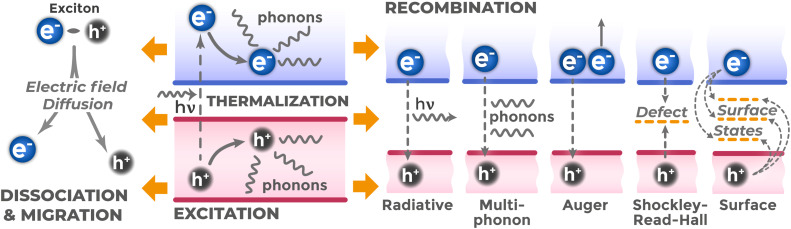
Main pathways of photogenerated electron–hole pair evolution: thermalization, exciton formation, its subsequent dissociation and carrier migration (useful pathway), as well as competing recombination processes (radiative, multiphonon, Auger, Shockley–Read–Hall, surface).

After photon absorption, an electron–hole pair is generated, which initially forms a bound state through Coulomb attraction – an exciton. Charge carriers in the exciton typically possess excess energy compared to the energy of corresponding band edges.^56^ This energy excess is quickly lost (within pico- or femtoseconds)^56^ in the thermalization process, when excited electrons and holes interact with the crystal lattice, emitting phonons, and relax to the CB bottom and VB top respectively (Fig. 6). After thermalization, exciton evolution is determined by competition between two main processes: dissociation into free charge carriers and direct recombination. If the exciton dissociates, the formed free electrons and holes can migrate in the semiconductor bulk through diffusion (caused by their concentration gradient) and drift (ordered motion under electric field action). Competing recombination processes occur at all stages. These processes annihilate electron–hole pairs and cause most charge carrier losses.

Recombination can be either radiative or non-radiative. In radiative recombination, energy is released as a light quantum. This process underlies the operation of LEDs and lasers^57^ and occurs more often in direct band gap semiconductors. In non-radiative recombination, the energy released during electron–hole pair annihilation is transferred to the crystal lattice as heat (multiphonon recombination)^58^ or to another charge carrier (Auger recombination).^59^ Non-radiative recombination often occurs through local energy levels in the band gap created by crystal structure defects or impurity atoms (Shockley–Read–Hall mechanism^59,60^).

Surface recombination presents another loss channel.^61^ The physical surface of a semiconductor crystal contains surface states due to lattice periodicity disruption, adsorbed particles and other defects. These surface states act as recombination centers for non-equilibrium charge carriers reaching the surface.

### 2.2. The semiconductor–electrolyte interface and electrochemical behavior

When a semiconductor contacts an electrolyte, charge transfer occurs across the interface until thermodynamic equilibrium is established, which modifies the energy structure of the near-surface region. Upon contact of these two phases, charge redistribution begins across the interfacial boundary.^62^ This spontaneous charge transfer continues until thermodynamic equilibrium is established, where the electrochemical potential of electrons in the semiconductor (its Fermi level) equalizes with the averaged electrochemical potential in the contact layer of the electrolyte.^39^ If the electrochemical potential of electrons in the semiconductor differs from that in the electrolyte, the system is in a non-equilibrium state, and electrons will spontaneously transfer from the phase with higher potential to the phase with lower potential.^63^ If a dominant redox system (for example, Cr(vi)/Cr(iii)) is present in the electrolyte, its equilibrium redox potential will determine this effective electrochemical potential of the electrolyte.

**Fig. 7:**
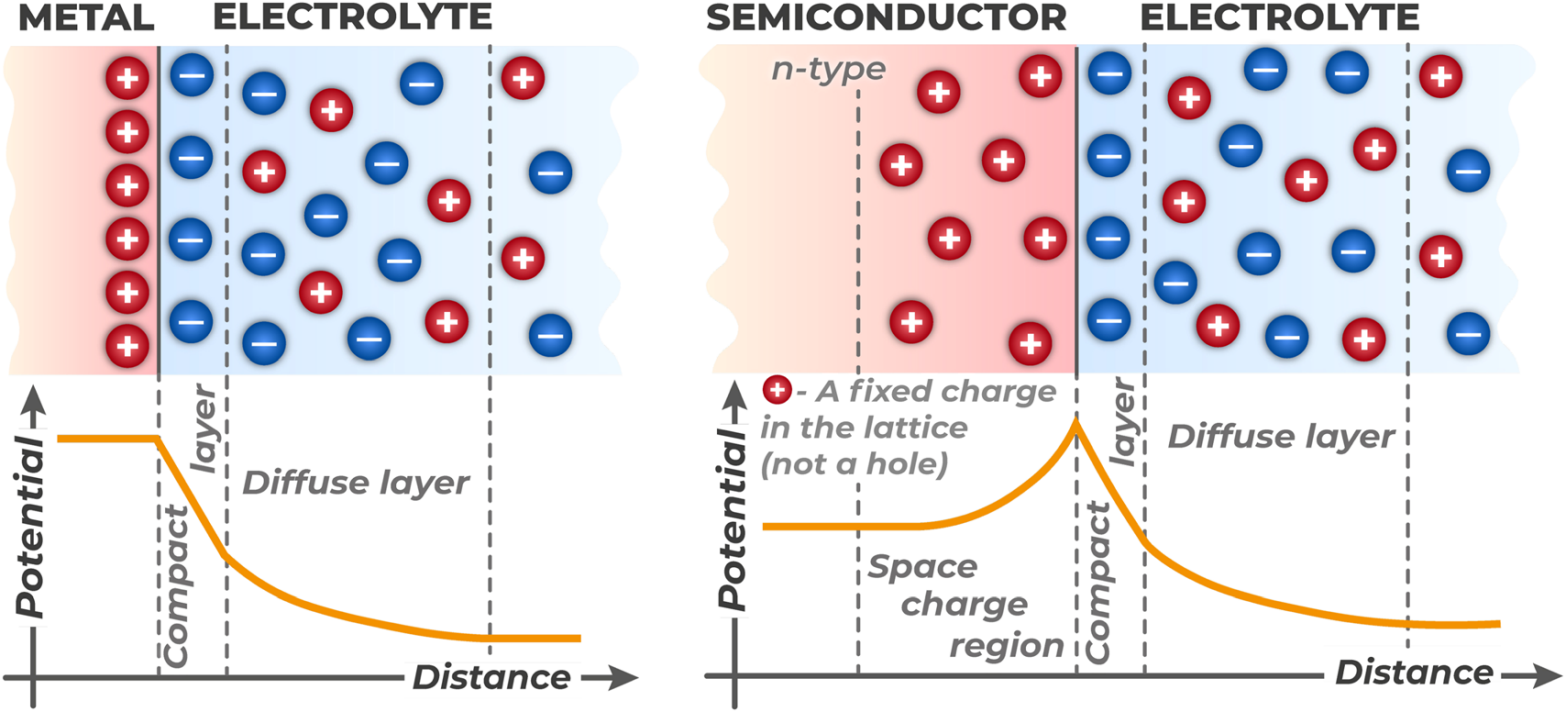
Comparison of electric double layer (EDL) structure and potential distribution at the metal/electrolyte and semiconductor/electrolyte interfaces.

As a result of such charge redistribution, an electric double layer (EDL) forms at the interface. The EDL structure differs significantly for metals and semiconductors due to substantial differences in free charge carrier concentration,^39^ as illustrated in Fig. 7. In metals, which possess high electron concentration, excess charge is localized in a very thin layer (Thomas–Fermi screening length for metals like platinum or gold is 0.5 Å (ref. 64)) directly at the metal surface. On the electrolyte side, this charge is compensated by a layer of ions (Helmholtz layer and diffuse layer). Thus, in metals, all potential drop occurs in the electrolyte part of the EDL, while inside the metal itself there is no electric field.

For semiconductors, the situation is different. Due to significantly lower concentration of free charge carriers (electrons and holes), the electric field cannot be effectively screened at the surface itself. Consequently, excess charge on the semiconductor side is distributed in its near-surface region at a significant distance from the interface (for silicon, the depletion layer thickness can be 35 nm (ref. 65)), forming a space charge region (SCR). This layer arises due to depletion or, conversely, enrichment of the near-surface region with mobile charge carriers and is characterized by the presence of uncompensated charge – either ionized dopant atoms (in the case of a depleted layer) or excess mobile carriers (in the case of an enriched layer). The SCR charge in the semiconductor is compensated by charge formed on the electrolyte side in the form of a Helmholtz layer and diffuse layer of ions.

The formation of the EDL upon semiconductor contact with electrolyte is a rapid transient process that occurs within tens of milliseconds after phase contact^66^ and brings the system to thermodynamic equilibrium. The energy structure of the interface established determines all subsequent photoinduced phenomena. The presence of an electric field in the space charge region leads to bending of energy bands (VB and CB) in the near-surface region of the semiconductor relative to their position in the bulk.^51,62^ The physical nature of this phenomenon is that the electric field creates an electrostatic potential gradient *φ*(*x*) in the direction from the surface into the material. Since the potential energy of an electron equals (−*eφ*), where *e* is the elementary charge,^39^ potential change leads to corresponding change in energy of all electronic states. In energy diagrams, this manifests as smooth curvature of VB and CB edges, which remain horizontal in the material bulk (far from the surface). The direction and magnitude of this bending depend on the semiconductor type (n- or p-type), its doping level, and the potential difference at the interface with the electrolyte.

**Fig. 8:**
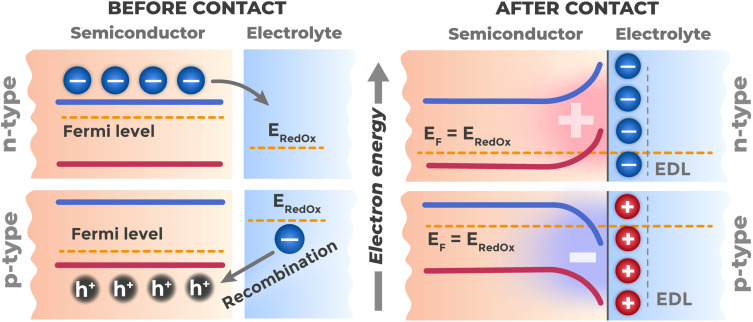
Formation of energy band bending at the interface of n- and p-type semiconductors with electrolyte as a result of alignment of their Fermi levels (*E*_F_) with the electrolyte redox potential (*E*_RedOx_).

When electrons transfer from an n-semiconductor to the electrolyte, local charge redistribution occurs. In the semiconductor, positively charged donor ions remain uncompensated, forming a charged space charge region.^62^ Simultaneously, in the electrolyte near the interface, negative charges accumulate – either excess anions, or reduction products, or electrons localized on surface states. The electron transfer process is self-limiting: as positive charge accumulates in the semiconductor, an electric field arises that prevents further electron transfer. Equilibrium is established when the electrostatic potential created by separated charges exactly compensates the initial difference in electrochemical potentials of the phases. This leads to formation of an SCR depleted of electrons and enriched with positively charged donor ions, and, consequently, to upward band bending, as shown in Fig. 8. For a p-semiconductor in an analogous situation, holes transfer to the electrolyte (which is equivalent to electron transfer from electrolyte to semiconductor), creating an SCR depleted of holes and enriched with negatively charged acceptor ions, causing downward band bending, also depicted in Fig. 8. In intrinsic semiconductors, where electron and hole concentrations are equal and the Fermi level is in the middle of the band gap, band bending will be less pronounced and its direction depends on specific electrolyte properties.

The electric field established in the SCR spatially separates photogenerated electron–hole pairs upon illumination: in n-type, holes move along the field direction toward the interface to participate in electrochemical reactions, while electrons move against the field into the semiconductor bulk toward the ohmic contact.^67^ In p-type semiconductors, the separation direction is opposite: electrons move along the field direction toward the surface to participate in reduction reactions, while holes move against the field into the material bulk toward the ohmic contact.

There exists a certain electrode potential at which band bending at the semiconductor/electrolyte interface is absent, *i.e.*, energy bands remain flat up to the surface. This potential is called the flat band potential (*U*_fb_)^39^ and is an important electrochemical characteristic of a specific semiconductor/electrolyte interface. Knowledge of *U*_fb_ serves as a reference point for controlling charge separation efficiency: at potentials more positive than *U*_fb_ (for n-type), band bending and built-in field strengthen, improving separation of photogenerated carriers, while at more negative potentials the field weakens or may change direction, leading to reduced photoresponse.

**Fig. 9:**
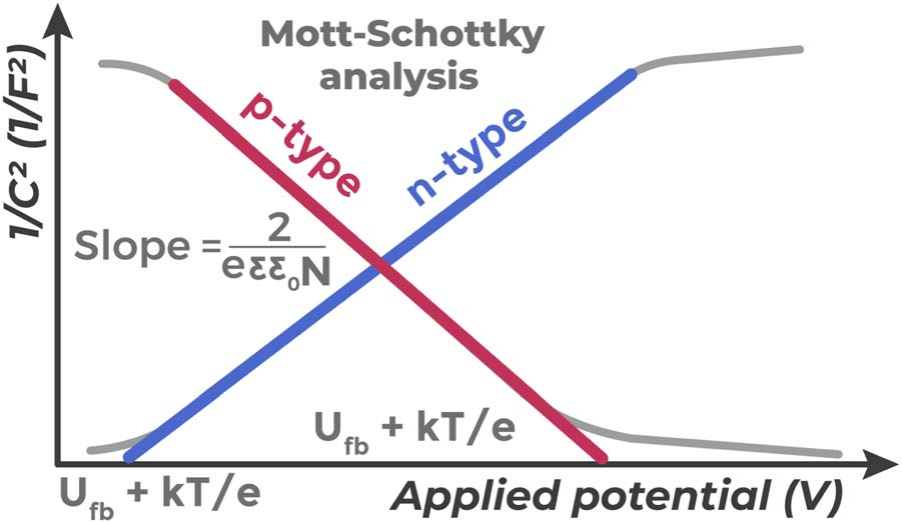
Principle of Mott–Schottky analysis. Plot of 1/*C*^2^ dependence on applied potential (*V*) for n- and p-type semiconductors for determining impurity concentration and flat band potential (*V*_fb_).

For experimental determination of *U*_fb_ and other characteristics of the semiconductor/electrolyte interface, the Mott–Schottky test is widely used, whose principle is presented in Fig. 9. This method presents a type of electrochemical impedance spectroscopy and is performed in a standard three-electrode cell, where the capacitance of the space charge region (*C*) is measured as a function of applied potential (*V*).

Often the analysis is simplified by conducting measurements at one, pre-selected frequency.^68^ However, such an approach can distort results: due to frequency dispersion phenomena,^69^ the graph slope can strongly depend on frequency choice. Therefore, to confirm data correctness, it is extremely important to either justify the working frequency choice or demonstrate that graphs for different frequencies converge at one flat band potential point.^68^ The theoretical basis of the method is the Mott–Schottky equation:^70^1
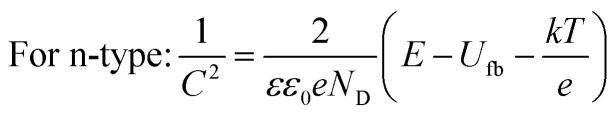
2
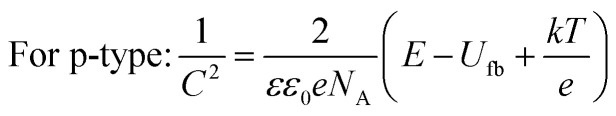
where *C* is the SCR capacitance, *e* is the electron charge, *ε* is the relative dielectric permittivity of the semiconductor, *ε*_0_ is the vacuum dielectric permittivity, *N* is the majority charge carrier concentration (donors for n-type or acceptors for p-type), *E* is the applied potential, *U*_fb_ is the flat band potential, *k* is the Boltzmann constant, *T* is temperature.

A plot of 1/*C*^2^*versus V* dependence is constructed, which in the ideal case presents a straight line. From this dependence, the following can be determined: semiconductor conductivity type (positive slope for n-type, negative for p-type), majority charge carrier concentration (from line slope), and flat band potential (from extrapolation to intersection with potential axis at 1/*C*^2^ = 0). Although Mott–Schottky analysis is one of the most widely used methods for determining semiconductor photoelectrode parameters, interpretation of obtained data should be approached with great caution.^68,71^

Photoelectrode efficiency is directly related to how successfully photogenerated charge carriers can be separated and directed to participate in useful processes before they recombine. Exciton dissociation occurs primarily due to the built-in electric field in the SCR, when field forces acting on electron and hole in opposite directions exceed their binding energy.^72^ Accordingly, for separation, carriers should be either generated directly in the SCR, where strong electric field ensures their separation, or in the semiconductor bulk at a distance from the SCR boundary, from where they can reach the separation region through diffusion.^73^ The critical parameter here is the diffusion length (*L*) – the average distance a charge carrier travels in the material through diffusion during its lifetime before recombination:^73^

**Fig. 10:**
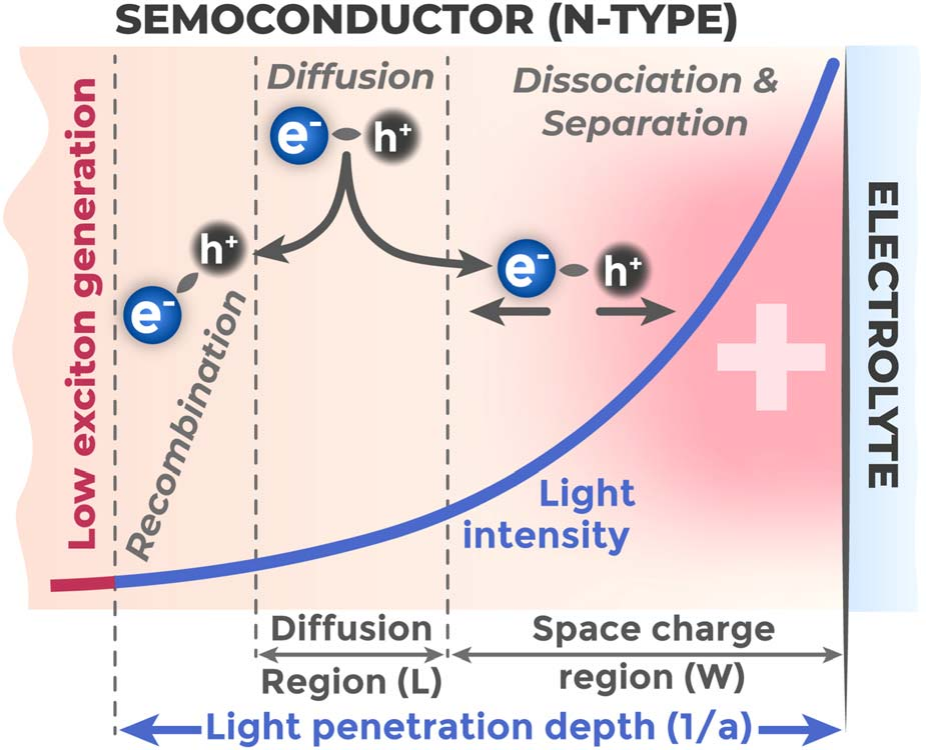
Relationship between light penetration depth (1/*α*), space charge region width (*W*), and carrier diffusion length (*L*).

3 *L*_D_ = (*Dτ*)^1/2^

where *D* is the charge carrier diffusion coefficient, *τ* is the charge carrier lifetime before recombination. Carrier collection efficiency also depends on light penetration depth (1/*α*) – the distance over which incident light intensity decreases by a factor of *e*, since this quantity determines the main electron–hole pair generation region.^74^ Carriers generated by light deeper than *L* + *W* (where *W* is the SCR thickness) cannot diffuse to the effective separation region and will recombine in the bulk. Therefore, for optimal carrier collection, it is necessary to match light penetration depth with the total thickness of SCR and minority carrier diffusion length, as visualized in Fig. 10.

The photoelectrode operation mode (photoanode or photocathode) is determined by a combination of factors: semiconductor type, applied external potential, and electrolyte thermodynamic characteristics. An n-semiconductor can operate as a photoanode when a sufficiently positive potential is applied (relative to *U*_fb_), which strengthens upward band bending and ensures hole movement to the surface for oxidation reactions. At more negative potentials, the same n-semiconductor can function as a photocathode.

Photogenerated charge carriers that reach the semiconductor–electrolyte interface can participate in electrochemical reactions. On n-semiconductors operating in photoanode mode, holes move to the surface and oxidize electrolyte components, generating anodic photocurrent. This photocurrent is faradaic – its magnitude directly relates to the reaction rate on the photoelectrode surface. For circuit closure, conjugated reactions must occur on both electrodes: oxidation at the photoanode and reduction at the counter electrode. Each electrochemical reaction corresponds to one electron passing through the external circuit. On p-semiconductors functioning as photocathodes, electrons move to the surface under illumination.

Despite the SCR field promoting charge separation, surface recombination remains a significant loss channel. If charge transfer to electrolyte components is slow due to poor reaction kinetics, carrier concentration at the surface increases, raising recombination probability with majority carriers still present in the near-surface region. For some semiconductor materials, especially with narrow band gap or chemically unstable ones, photocorrosion may occur^75^ (oxidation or reduction of the electrode material by photogenerated carriers). Photocorrosion is thermodynamically possible if hole energy in the VB can oxidize the electrode material, or electron energy in the CB can reduce it. This leads to photoelectrode degradation over time. Managing these competing processes is important for developing stable and efficient photoelectrochemical systems.

### 2.3. Photoelectrochemical cell design and detection principles

PEC sensor performance depends on proper design of the measurement cell, which must provide suitable conditions for photoexcitation, charge transfer, and electrochemical chromium(vi) detection reactions. PEC cells differ from standard electrochemical ones by including an optically transparent window made of fused quartz,^76^ which allows light penetration to the photoactive electrode.

**Fig. 11:**
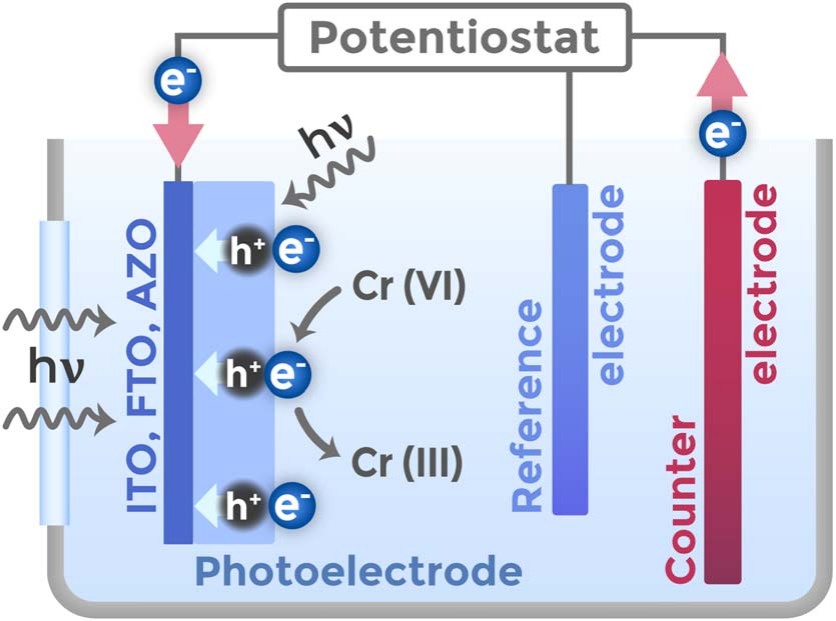
Schematic diagram of a three-electrode photoelectrochemical cell for Cr(vi) detection.

The three-electrode configuration includes a working electrode in the form of photoactive semiconductor material on a conductive substrate, a counter electrode made of inert material, and a reference electrode for precise potential control – Fig. 11. The cell design provides two possible modes of photoelectrode illumination: front illumination through the electrolyte and back illumination through the substrate. The choice between front and back illumination determines substrate requirements and affects sensor efficiency. Back illumination requires the use of transparent conducting oxides as substrates, such as ITO, FTO, AZO.^77,78^ Front illumination provides maximum charge carrier generation near the electrode surface, which reduces requirements for hole diffusion length but increases requirements for electron transport through the film thickness. Back illumination creates a charge carrier gradient with maximum at the back surface, requiring efficient hole transport.^77^

As previously discussed, semiconductors generate charge carriers only when photon energy exceeds the band gap. Selecting appropriate illumination therefore requires experimental determination of this critical parameter. Band gap characterization traditionally employs UV-vis diffuse reflectance spectroscopy (DRS) data analyzed using the Tauc equation:^79^

4 (*αhν*)^1/*γ*^ = *B*(*hν* − *E*_g_)

where *α* is the absorption coefficient, *h* is Planck's constant, *ν* is photon frequency, *E*_g_ is the band gap energy, *B* is a material constant, and *γ* depends on the nature of electronic transitions (*γ* = 1/2 for direct transitions, *γ* = 2 for indirect transitions). Since the Tauc method requires absorption coefficient data, the measured reflectance spectra must be converted using the Kubelka–Munk transformation to extract the absorption information from reflection measurements.^79^

Plotting this relationship yields a linear region whose extrapolation to the energy axis provides the band gap value. However, this straightforward analysis becomes problematic for doped, defective, or surface-modified materials. These systems often display continuous absorption below the fundamental edge due to localized states within the forbidden gap, creating sub-threshold absorption that can lead to underestimated band gap values if not properly accounted for.^79^

Indiscriminate use of high-energy UV illumination represents suboptimal practice without prior spectroscopic characterization. While UV photons carry sufficient energy for most semiconductors, excessive photon energy introduces complications. Surface reflectance may increase at short wavelengths due to rising refractive indices,^80^ reducing effective light penetration. Moreover, at very high energies, the density of electronic states available for useful transitions may decrease,^81^ reducing absorption efficiency.

Contemporary PEC sensor research employs diverse illumination strategies: xenon sources^82,83^ for broad visible spectrum coverage, mercury vapor lamps for controlled UV exposure,^84^ LED arrays providing discrete wavelengths,^85,86^ standardized solar simulators (AM 1.5G),^87^ and tungsten-halogen lamps for continuous broadband radiation.^88^ While the common 100 mW cm^−2^ intensity standard enables inter-laboratory comparisons, researchers must recognize that identical power densities deliver vastly different photon fluxes depending on spectral content.

Intensity decreases proportionally to the square of distance, and changing the angle of incidence affects the effective irradiation area and penetration depth.^89,90^ For complete PEC sensor characterization, it is necessary to conduct photocurrent measurements as a function of individual light wavelengths using a monochromator. This approach determines which wavelengths most efficiently generate photocurrent and allows optimization of spectral response to chromium(vi).

Electrolyte selection must provide sufficient solution conductivity for efficient ion transfer between electrodes. To ensure conductivity, salts of ions stable in a wide potential range can be used, such as sodium sulfate,^91^ which do not participate in side electrochemical reactions and do not affect target analyte detection. Precise pH control is often required, so buffer systems are used,^85^ since medium acidity affects the form of Cr(vi) existence in solution, electrochemical reaction kinetics, and photoelectrode material stability. Additionally, some systems use sacrificial reagents such as ascorbic acid,^92^ which act as hole acceptors and enhance charge separation. These reagents are added to the solution with the analyte, and measurements are taken immediately after illumination begins, so reagent consumption during detection is minimal. However, in continuous monitoring scenarios, such as real water monitoring, sacrificial agent depletion could cause photocurrent decline. At the same time, continuous monitoring with such sensors would be impractical due to the need to add large quantities of these agents to natural waters.

**Fig. 12:**
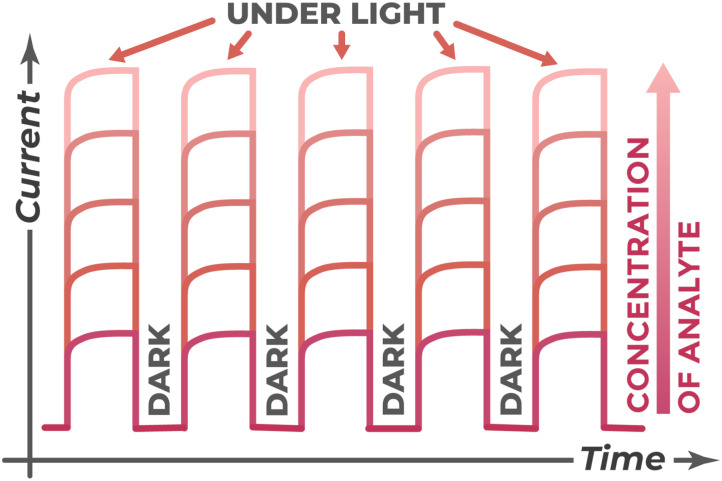
Typical chronoamperogram in intermittent illumination mode, demonstrating photocurrent increase with increasing analyte concentration.

Practical measurements are conducted in potentiostatic mode using a potentiostat. Application of controlled external potential allows control of charge carrier movement, maintenance of stationary measurement conditions, and ensures high reproducibility of results. For photoelectrochemical sensors, the key factor is the change in photocurrent magnitude in the presence of and depending on target analyte concentration. To obtain analytical signal and construct calibration curves, the chronoamperometry method with intermittent illumination is widely used,^83,93^ whose typical response is shown in Fig. 12.

The essence of the method consists in periodic switching on and off of the light source at fixed potential. In darkness, only insignificant background dark current is registered. When light is turned on, a sharp current jump occurs due to charge carrier generation and separation – this is the measured photocurrent. The analytical signal is the difference between current in light and in darkness. The magnitude of this signal is determined for an entire range of analyte concentrations, allowing construction of a calibration curve.

## 3. Materials strategies for photoelectrochemical sensing

Developing high-performance sensing systems demands sophisticated materials design. This section examines materials strategies for developing photoelectrochemical sensors for hexavalent chromium detection. Researchers use both single-component systems and multifunctional architectures. Various materials approaches can improve sensor performance. This section explores single-phase semiconductor systems and their limitations, then discusses semiconductor heterostructures for charge separation and spectral sensitivity expansion. We examine quantum dots and plasmonic nanostructures for optical tuning and light harvesting. The discussion covers engineering of conductive transport networks for charge transfer and molecular surface modification strategies for improved selectivity.

### 3.1. Single-phase semiconductor systems for Cr(vi) detection

Cr(vi), due to its high redox potential, is an effective electron acceptor and is thermodynamically capable of interacting with photogenerated charge carriers in most semiconductor materials. The thermodynamic possibility of such interaction is determined by the energy relationship between the semiconductor CB bottom and the redox potential of the Cr(vi)/Cr(iii) couple. When the energy level of electrons in the CB exceeds the Cr(vi) reduction potential, the charge transfer process becomes thermodynamically favorable. Since Cr(vi) is in its highest stable oxidation state, it can only act as an electron acceptor. Accordingly, from a theoretical standpoint, p-type semiconductors operating in photocathode mode should be most suitable for Cr(vi) detection. In such systems, photogenerated electrons are directed by the built-in electric field directly to the electrolyte interface, where they can reduce Cr(vi) ions to Cr(iii). However, n-type semiconductors functioning as photoanodes are more commonly used, where holes move to the surface and electrons move into the material bulk.

**Fig. 13:**
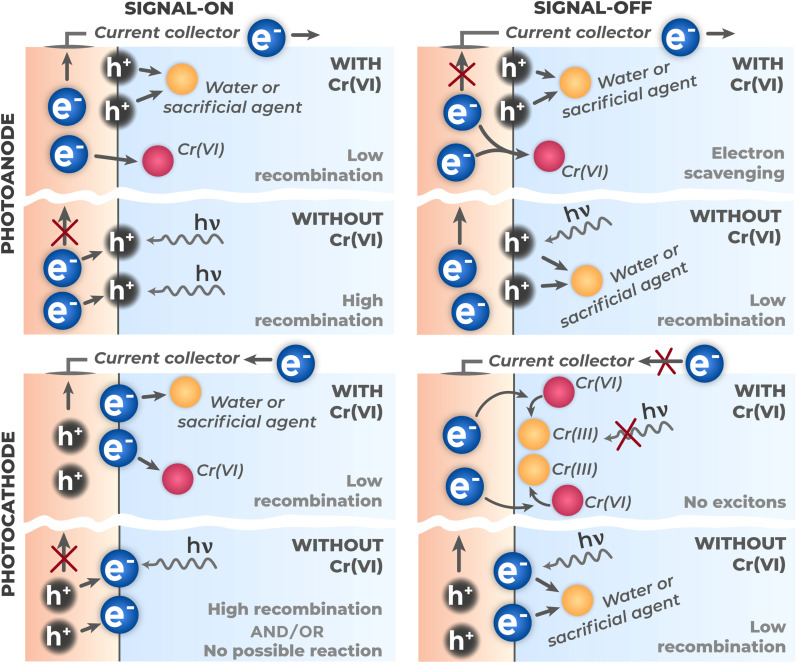
Schematic presentation of main Cr(vi) detection mechanisms on photoanodes and photocathodes: “signal-on” and “signal-off”.

The electrode operation mode (photoanode or photocathode) is determined not only by the material conductivity type and electrolyte redox potential, but also by the applied external potential. Controlled potential bias allows control of energy band bending and, consequently, the direction of charge carrier movement. The analytical signal results not only from direct Cr(vi) interaction with charge carriers, but also from the balance of all kinetic processes occurring at the electrode surface. Depending on the dominant mechanism, Cr(vi) presence can lead to two fundamentally different types of response: photocurrent enhancement (signal-on), when photocurrent increases with increasing analyte concentration, and its attenuation (signal-off), when photocurrent decreases instead. Fig. 13 presents a comparison of main detection mechanisms for both photoanode and photocathode configurations.

The photocurrent enhancement mechanism (signal-on) is based on Cr(vi) ability to effectively suppress electron–hole pair recombination through selective scavenging of photogenerated electrons. A classic example of such mechanism implementation is the system based on bismuth vanadate (BiVO_4_)^91^ functioning as a photoanode. In the absence of Cr(vi), a significant portion of electrons recombines with holes, limiting the overall quantum yield of the process and, consequently, the measured photocurrent. Upon Cr(vi) addition to the electrolyte, chromium ions act as electron traps, rapidly scavenging them and thereby preventing recombination. As a result, a larger fraction of photogenerated holes successfully reaches the electrode surface, leading to increased anodic photocurrent proportional to Cr(vi) concentration.

The photocurrent attenuation mechanism (signal-off) is realized when Cr(vi) competes for photogenerated electrons with processes that form the main analytical signal. Unlike the signal-on mechanism, where electron scavenging promotes charge separation, interaction with Cr(vi) in this case decreases measured photocurrent. An example of such behavior is highly ordered arrays of titanium dioxide nanotubes (TiO_2_-NTAs) functioning as photoanodes under UV illumination.^84^ In this system, TiO_2_ generates a certain baseline anodic photocurrent in the absence of analyte, caused by oxidation of electrolyte components (*e.g.*, water or trace amounts of organic impurities) by photogenerated holes on the photoanode surface. Upon Cr(vi) addition, chromium ions act as scavengers of photogenerated electrons, diverting them from processes that ensure the main photocurrent.

Although in both cases Cr(vi) ions consume photogenerated electrons, this leads to opposite results – photocurrent increase or decrease. The difference is determined by the kinetic balance between charge carrier recombination rate and their transfer rate to chromium ions. In the signal-on mechanism, characteristic of photoanodes with initially high recombination rate, Cr(vi) appearance creates a faster pathway for electrons, reducing recombination. As a result, a larger fraction of holes reaches the surface and participates in the main oxidation reaction, requiring flow of more electrons to the external circuit and, consequently, leading to increased measured anodic photocurrent. In the signal-off mechanism, typical of systems with initially low recombination rate, photocurrent in the absence of analyte is already stable and high. Cr(vi) addition creates a competing pathway that diverts electrons initially intended for the external circuit.

The signal-on mechanism can also be implemented in photocathode mode. In this scenario, the initial cathodic photocurrent in the absence of analyte is very low or absent altogether, due to high electron–hole pair recombination rate at the surface or in the semiconductor bulk, or absence in the background electrolyte of any components that are thermodynamically capable of accepting electrons from the photocathode surface. Cr(vi) ions added to solution act as acceptors for photogenerated electrons. This creates a charge transfer channel across the semiconductor–electrolyte interface and competes with the recombination process, suppressing it. Electrons that previously recombined rapidly can now participate in electrochemical reactions, producing cathodic photocurrent that increases with Cr(vi) concentration. Sensors based on bismuth oxyiodide decorated with metallic bismuth nanoparticles (Bi/BiOI) demonstrate this mechanism.^94^ It is important to note that this work does not fully belong to single-phase semiconductors, since metallic bismuth introduction is aimed at creating surface plasmon resonance effect, which will be discussed below. Nevertheless, the mechanisms discussed here are universal and applicable to all semiconductor structure architectures considered further.

The signal-weakening mechanism presents a special case of signal-off response, where photocurrent decrease is caused not by kinetic competition for charge carriers, but by physical blocking of the electrode active surface by electrochemical reaction products with the analyte. Classic examples of such approach implementation are photocathodes based on copper oxide (CuO)^87^ and nickel oxide (NiO)^95^ films, presenting p-type semiconductors. In these systems, photogenerated electrons are directed by the built-in electric field to the electrolyte interface, where they effectively reduce Cr(vi) to Cr(iii). The formed Cr^3+^ ions under neutral or weakly alkaline conditions hydrolyze and precipitate on the electrode surface as insoluble chromium hydroxide Cr(OH)_3_. The accumulating precipitate layer blocks light access to the photoactive surface, limits reagent diffusion to active sites, and increases charge transfer resistance across the interface. These effects progressively reduce the initial cathodic photocurrent.

Single-phase semiconductor systems have several limitations. Photogenerated electron–hole pair separation efficiency is low because separation competes with bulk and surface recombination processes. Spectral sensitivity tuning options are limited since band gap width determines the absorbed light range. Materials must balance chemical stability with photocatalytic activity. These limitations drive the use of more complex heterostructural architectures. Such architectures allow targeted control of charge carrier flows, spectral sensitivity expansion, and energy optimization while maintaining material stability.

### 3.2. Semiconductor heterostructures and junction engineering

Upon forming contact between two semiconductors, similar to what occurs at the interface with electrolyte, the fundamental process is the alignment of electrochemical potentials (Fermi levels). This process is initiated by charge transfer across the interface until thermodynamic equilibrium is established. The general consequence of this transfer, as in the case with electrolyte, is the formation of space charge regions and energy band bending in interfacial regions.^52^ As a result, an internal electric field arises that can influence charge carrier movement.

**Fig. 14:**
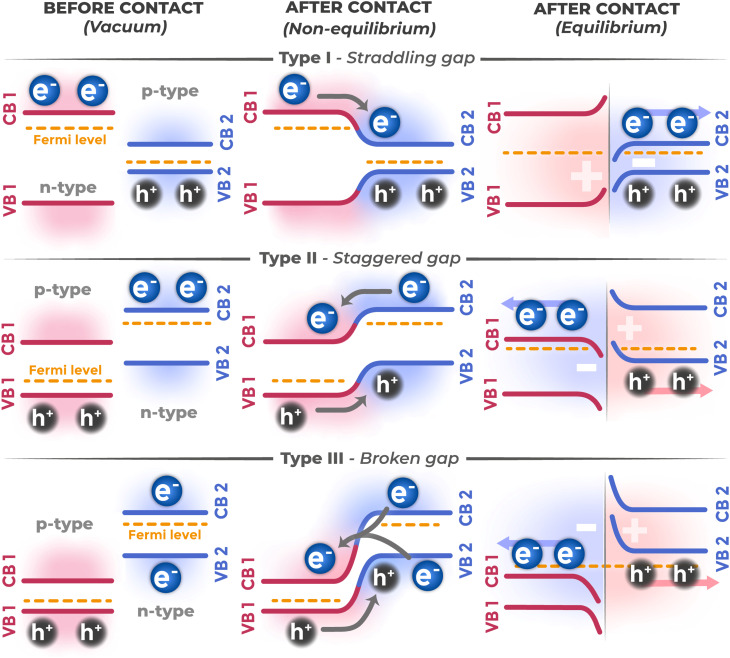
Classification of semiconductor heterojunctions depending on mutual arrangement of energy bands.

However, despite this phenomenological commonality, the nature and functionality of the heterojunction have fundamental differences. The potential drop here is distributed between two SCRs (one in each semiconductor). Charge transfer across the heterojunction is predominantly electronic in nature, without chemical transformations, while at the electrolyte interface, charge transfer across the boundary is coupled with electrochemical reactions. Depending on the mutual arrangement of valence band and conduction band edges at the interface, heterojunctions are typically classified into three main types, as shown in Fig. 14. This classification allows prediction of the main direction of photogenerated charge carrier migration and, consequently, assessment of heterostructure suitability for various applications.

#### 3.2.1. Type I (straddling gap)

In a type I heterojunction, the band gap of one semiconductor is completely within the band gap of another. As a result of such band arrangement, both photogenerated electrons and holes energetically tend to localize in the same narrow-gap semiconductor. Such spatial colocalization of carriers of both signs leads to enhanced recombination.^96^

#### 3.2.2. Type III (broken gap)

In a type III heterojunction, the conduction band of one semiconductor is lower in energy than the valence band of another. This energy structure creates overlapping band states, enabling interband tunneling. Electrons can tunnel from the valence band of one material directly into the conduction band of another, and *vice versa*, bypassing the band gap. However, tunneling effects create high dark leakage currents and complicate carrier transfer control.^97^

#### 3.2.3. Type II (staggered gap)

Type II heterojunction is most demanded for photoelectrochemical applications since it provides effective spatial separation of photogenerated charge carriers through the classical mechanism of migration along energy gradient. In this configuration, semiconductor energy bands are shifted relative to each other such that after electron–hole pair generation, electrons migrate to the semiconductor with lower CB bottom potential, and holes migrate to the material with higher VB top potential. This spatial separation suppresses recombination and increases charge carrier lifetime.

An example of such mechanism is the MoS_2_/BiOI composite,^83^ where electron transfer from MoS_2_ conduction band to BiOI conduction band is proposed, and holes transfer from BiOI valence band to MoS_2_ valence band. The effectiveness of this heterostructure approach is demonstrated by improvements over individual components – the MoS_2_/BiOI-5 composite exhibited lower photoluminescence intensity compared to pure BiOI. Additionally, it showed the lowest charge transfer resistance among all tested materials, confirming enhanced electron transfer kinetics. Since radiative recombination is one of the loss channels in photoelectrochemical systems, reduced luminescence intensity indicates effective recombination suppression and, consequently, increased quantum yield of photoelectrochemical response.^98^

Similarly, in the CuS/Bi_2_MoO_6_ system,^82^ a type-II mechanism is realized where narrow-gap CuS acts as a photosensitizer effectively absorbing visible light, and photogenerated electrons and holes are separated between heterostructure components. The heterostructure formation significantly extends charge carrier lifetime from 1.64 ns for pure Bi_2_MoO_6_ to 2.03 ns for composite. Electrons accumulated on the Bi_2_MoO_6_ conduction band reduce Cr(vi) to Cr(iii), providing signal-on sensor response.

The most powerful variant of type II heterojunction implementation is the contact of p- and n-type semiconductors. When they are joined, diffusion of majority carriers and their recombination at the interface form a depleted layer with strong internal electric field, providing effective separation of photogenerated electron–hole pairs. An example is the p-BiOI/n-CN system,^93^ where electrons transfer from BiOI conduction band to carbon nitride conduction band, and holes migrate in the opposite direction. The effectiveness of this p–n heterojunction is evidenced by the optimal BiOI/CN-34% composite showing the fastest photoluminescence decay (1.3 ns compared to 1.9 ns for BiOI and 2.3 ns for CN) and the lowest charge transfer resistance. Faster photoluminescence decay indicates that photogenerated electrons and holes are rapidly separated or transferred to different components before they can recombine radiatively. Electrons accumulated on carbon nitride conduction band then reduce Cr(vi), providing sensor response. A similar type-II mechanism with p–n interface participation is realized in the Pb_5_S_2_I_6_–PDA/TiO_2_ composite,^85^ where p-type Pb_5_S_2_I_6_ serves as photosensitizer, and electron transfer to n-TiO_2_, mediated by polydopamine, provides effective charge separation.

Although p–n type heterojunctions within the type-II mechanism theoretically provide the most effective separation of photogenerated charges, other types of heterointerfaces are also actively investigated in photoelectrochemistry, including type I and III transitions.^99–102^ This is explained by the fact that effective functioning of photoelectrochemical systems is possible without maximum spatial charge carrier separation provided proper material architecture and surface process optimization, as shown by examples in Section 3.1.

Reduction of recombination losses can be achieved by alternative pathways: optimization of synthesis methods for defect tuning,^103^ acceleration of surface reaction kinetics, corrosion protection, and selection of materials with suitable energy levels for specific redox processes.^104^ Such approach is demonstrated by the NiCo-LDHs/TiO_2_-NTAs system,^105^ where nickel–cobalt layered double hydroxides act as cocatalyst for water oxidation reaction on the photoanode surface, accelerating hole consumption and thereby reducing recombination probability. If interfacial electron transfer rate exceeds their recombination rate with holes, the system demonstrates high quantum efficiency regardless of charge spatial separation degree.

Practical studies confirm the effectiveness of various heterostructural approaches. In SnS/Bi_2_MoO_6_ (ref. 106) and C–BiOCl/Bi_2_S_3_ (ref. 107) systems, heterostructure creation promotes increased efficiency of photogenerated carrier separation and transfer, achieved not only through spatial separation but also through improved kinetic and thermodynamic conditions for charge carrier collection.

Another advantage of heterostructural architectures is photosensitization – expansion of absorption spectrum into the visible region through inclusion of narrow-gap component that can absorb low-energy photons inaccessible to wide-gap material. For example, in the MoO_3_/Zn_0.7_Cd_0.7_S composite,^108^ the heterostructural material demonstrates broader visible light absorption region compared to individual components, provided by narrow-gap Zn_0.3_Cd_0.7_S acting as photosensitizer for wide-gap MoO_3_.

#### 3.2.4. Z-scheme heterojunctions

Although type II heterojunctions effectively solve the recombination problem, this is achieved at the cost of reduced redox ability of carriers. However, some heterostructures can simultaneously effectively separate charges and maintain high reactivity. To explain this phenomenon, which does not fit within the type II model, the Z-scheme model was proposed, whose principles are borrowed from natural photosynthesis processes.^109^

**Fig. 15:**
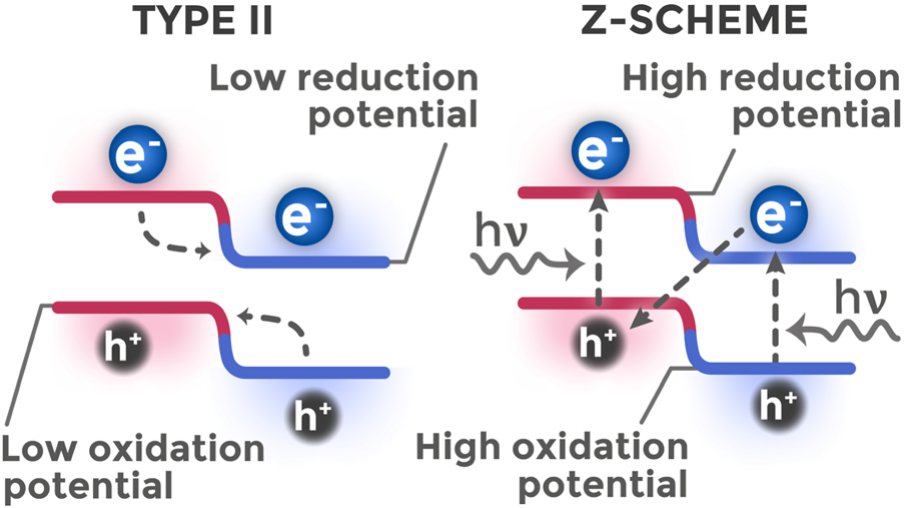
Comparison of charge transfer mechanisms in type II heterojunctions and Z-scheme.

The main idea of Z-scheme consists in selective recombination of charge carriers with less favorable redox potentials to preserve and accumulate carriers with stronger redox abilities.^110^ The redox ability of a carrier is determined by its potential, which corresponds to the energy band edge position where it is located after relaxation. Thus, an electron at the conduction band bottom with the most negative potential is a strong reducer, while an electron at the conduction band bottom with less negative potential has weaker reducing ability. Similarly, a hole at the valence band top with the most positive potential is a strong oxidizer, while a hole at the valence band top with less positive potential is a weaker oxidizer. Fig. 15 compares the charge transfer pathways in type II heterojunctions *versus* Z-scheme architectures.

In Z-scheme, recombination occurs of carriers with less favorable redox potentials (electron with weak reducing ability and hole with weak oxidizing ability), while carriers with strong redox potentials remain spatially separated in different semiconductors. To realize this mechanism, the kinetics of interfacial recombination of carriers with less favorable potentials must be significantly higher than kinetics of their transfer by type II mechanism.

In practice, this is achieved by two main pathways. In indirect Z-schemes, an electronic mediator (often a noble metal nanoparticle, *e.g.*, Au^111^) is introduced between two semiconductors, acting as a recombination center. Such architecture is realized in TiO_2_@C/Au/BiOI^92^ and ZnIn_2_S_4_/Au/In_2_S_3_ (ref. 112) systems. In direct Z-schemes, two semiconductors are in close contact, and recombination occurs directly at their interface, as in the ZnO/SnIn_4_S_8_ system, confirmed by theoretical calculations.^113^

A special approach to Z-scheme realization is demonstrated by systems where charge transfer pathway can be controlled by external influence. In the ZnO/MoS_2_ system, transition from type II mechanism to Z-scheme is induced by piezoelectric effect.^114^ Application of external mechanical stress created by solution stirring generates internal piezopotential in piezoelectric ZnO nanostructures. This potential modulates the energy diagram at the interface, kinetically promoting recombination *via* Z-scheme pathway. The effectiveness of such mechanism switching is confirmed by significant photocurrent increase – 3.3 times compared to stationary solution, clearly demonstrating significant superiority of Z-scheme in photogenerated charge carrier utilization efficiency in practice. It should nevertheless be noted that photocurrent increase here may also be related to improved mass transfer during stirring, which promotes more efficient analyte delivery to the electrode surface and overcomes diffusion limitations.

### 3.3. Quantum dots and plasmonic nanostructures

Besides creating heterostructures from bulk semiconductors, another powerful approach to improving PEC sensor characteristics is the use of nanoscale objects with unique optical and electronic properties. In this section, we will consider two key strategies: application of semiconductor quantum dots and plasmonic nanostructures of metals.

#### 3.3.1. Quantum dot systems (QDs)

Quantum dots (QDs) present semiconductor nanocrystals whose size is comparable to or smaller than the Bohr exciton radius – the characteristic average distance between electron and hole in bound state (exciton).^115^ When the physical size of a crystal limits carrier movement to such scales, the quantum size effect manifests.^116^ Its essence lies in the fact that continuous energy bands characteristic of bulk semiconductors split into a system of discrete, quantized energy levels, similar to energy levels in an individual atom.

From a fundamental standpoint, reducing nanocrystal size indeed leads to increased effective band gap width. This is explained by the “particle in a box” principle from quantum mechanics: the smaller the space in which a particle (in this case electron or hole) is confined, the higher its minimum possible (zero-point) energy.^117^ Energy increase for both electrons and holes leads to widening of the distance between ground electronic and hole levels, *i.e.*, to *E*_g_ increase.

The main advantage of this effect is the possibility to purposefully tune optical properties. This is not about always widening the band, but about selecting its width for optimal solution of a specific task. For example, one can tune the QD absorption edge so that it precisely matches the wavelength of an available light source (*e.g.*, cheap LED), ensuring maximum photon collection. Or one can shift band positions so that they have ideal redox potential for the target chemical reaction.

**Fig. 16:**
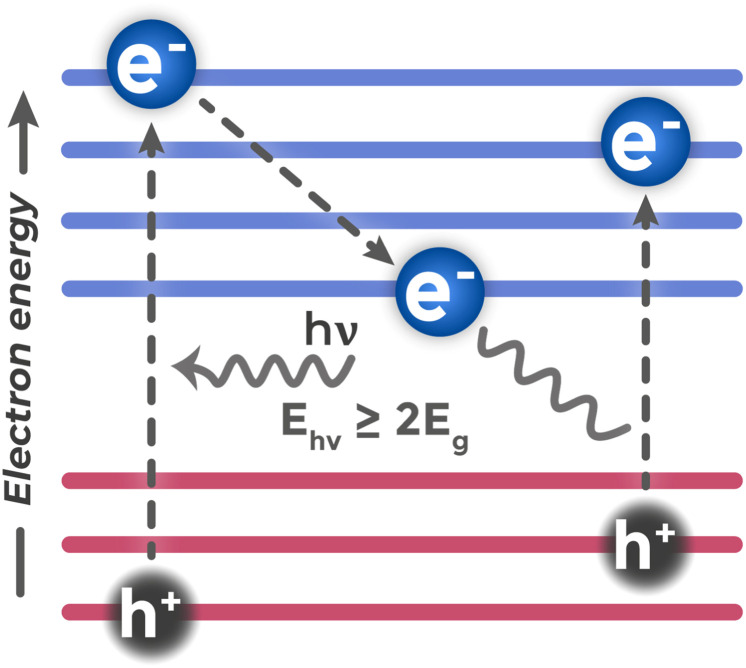
Electronic properties of quantum dots: discrete energy levels and multiple exciton generation mechanism.

Among other unique QD properties worth mentioning is multiple exciton generation (MEG).^118^ This process is possible when QD absorbs a photon with energy significantly exceeding its band gap (typically *E*_photon_ ≥ 2*E*_g_), as shown in Fig. 16.

The initially created electron–hole pair possesses large excess kinetic energy. In nanocrystals, this energy, instead of quickly dissipating as heat (phonons), can be used to excite a second (and sometimes subsequent^119^) electron from valence band to conduction band through impact ionization. Thus, one high-energy photon can generate several excitons, potentially increasing process quantum efficiency above 100%. MEG is fundamentally possible in bulk semiconductors too, but in quantum dots it occurs significantly more efficiently. In bulk materials, high-energy carriers quickly lose excess energy in thermalization processes. In QDs, size confinement modifies relaxation processes: discrete energy structure can create bottlenecks for phonon relaxation, increasing hot carrier lifetime. Additionally, in the small QD volume, Coulomb interaction between charge carriers is enhanced, increasing the probability of impact ionization processes.

QDs also often possess high luminescence quantum yield (ratio of emitted photons to absorbed ones), making them indispensable in areas such as optoelectronics or biomedicine,^120^ although in PEC sensors this property is usually not used, since exciton dissociation rather than radiative recombination is important there.

In work devoted to a system based on silver sulfide quantum dots (Ag_2_S QDs) and MnO_2_ nanosheets, narrow-gap Ag_2_S QDs are used as photosensitizer.^121^ They form with MnO_2_ a type II heterojunction. Upon light absorption, Ag_2_S QDs transfer photogenerated electron to MnO_2_ conduction band, while hole remains in QD valence band. Such spatial charge separation prevents their rapid recombination and provides baseline photocurrent. This photocurrent is enhanced upon addition of electron donor – ascorbic acid (AA) – to solution. AA molecules rapidly react with holes on QD surface, neutralizing them and more effectively suppressing recombination. The Cr(vi) detection mechanism here is based on indirect “signal-off” effect: Cr(vi) ions added to solution enter rapid chemical reaction with ascorbic acid, oxidizing it. As AA is consumed, its concentration at electrode surface drops, hole neutralization process slows down, recombination again begins to dominate, and measured photocurrent decreases.

In another work using lead sulfide quantum dots (PbS QDs), a direct detection mechanism is realized.^86^ Due to narrow band gap, these QDs efficiently absorb light in the blue visible spectrum region (wavelength 470 nm). The detection mechanism here is “signal-on”: Cr(vi) ions act as acceptors for photoelectrons generated in PbS QDs. This process directly competes with recombination, promoting charge separation and causing cathodic photocurrent enhancement.

#### 3.3.2. Plasmonic nanostructures

**Fig. 17:**
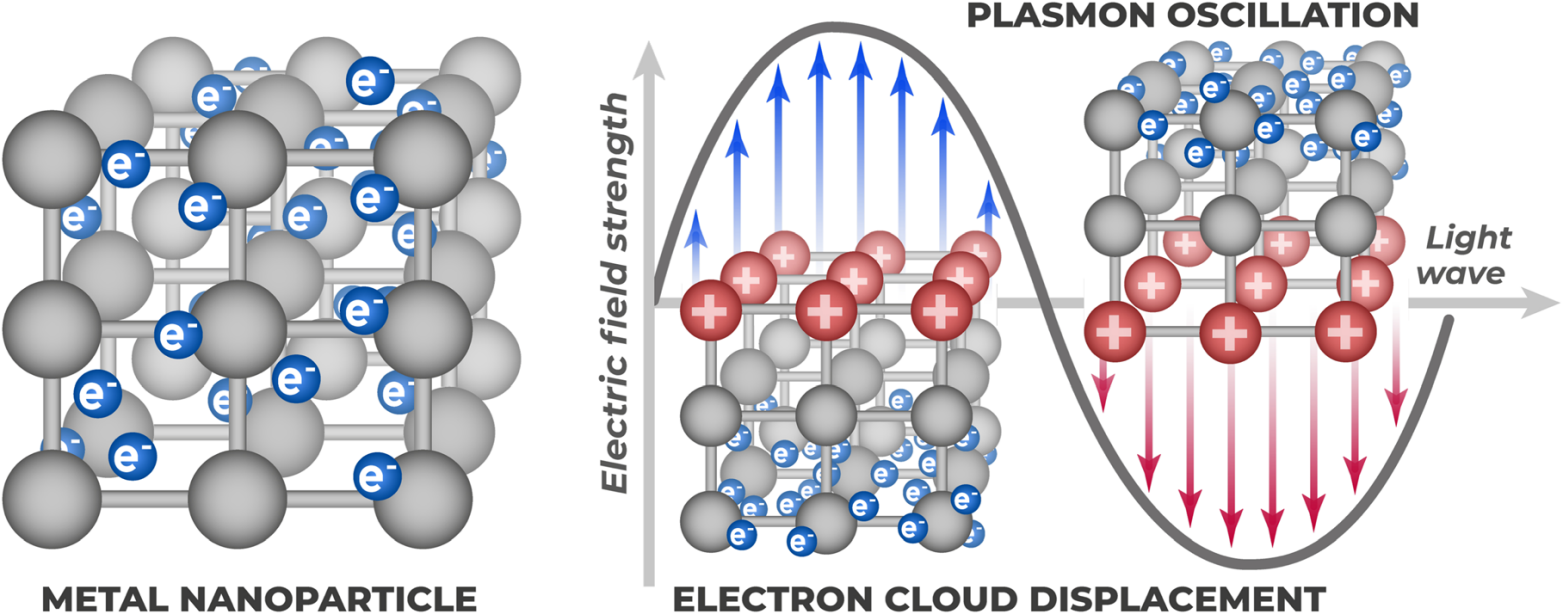
Principle of localized surface plasmon resonance (LSPR): collective coherent oscillations of free electrons in metallic nanoparticle.

Another strategy aimed at increasing light harvesting efficiency and charge carrier generation is the use of plasmonic nanostructures. The localized surface plasmon resonance (LSPR) phenomenon arises from light interaction with nanoparticles of materials possessing high density of free or quasi-free electrons (Fig. 17).

The essence of the phenomenon is as follows: electromagnetic light wave causes coherent collective oscillations of free electrons in nanoparticle relative to positively charged ionic lattice.^62^ A plasmon is a quantum of collective electron gas oscillations in conducting medium, a quasiparticle that describes bound state of electromagnetic field and oscillating electrons. The intrinsic frequency of these electronic oscillations is determined by fundamental metal properties – electron density and their effective mass.^122^ When incident light frequency matches this resonant frequency, energy transfer from light wave to electron cloud occurs.

Part of absorbed energy can then be scattered: oscillating electron gas itself becomes source of electromagnetic waves according to classical electrodynamics laws – any accelerated charge radiates electromagnetic waves, and collectively oscillating electrons are no exception. These secondary waves propagate in all directions, creating characteristic light scattering.

Although various metals are suitable for this (Cu, Al^123,124^), noble metals (Au, Ag) are preferred in PEC sensors.^125^ This is because their plasmonic resonances lie in convenient visible and near-IR spectral regions, and they possess high chemical stability under electrochemical conditions, unlike more easily oxidized metals. Recently, bismuth (Bi) is also actively investigated as a more accessible alternative.^126^

Incorporation of such plasmonic nanostructures into photoelectrode composition allows enhancement of its photoelectrochemical response through several mechanisms. One of them is local electromagnetic field enhancement. The enhancement mechanism consists in oscillating electrons creating dipole moment, which, in turn, generates its own electric field. This field adds to incident light field, with constructive interference occurring under resonant conditions.^127^

Also worth mentioning is plasmon-induced resonant energy transfer (PIRET) – excited plasmon energy is non-radiatively transferred to semiconductor through dipole interactions at distance, similar to Förster resonance energy transfer in molecular systems.^128,129^ In this case, exciton is directly created in semiconductor without intermediate radiation and photon absorption, which can be more efficient process than classical light scattering.

**Fig. 18:**
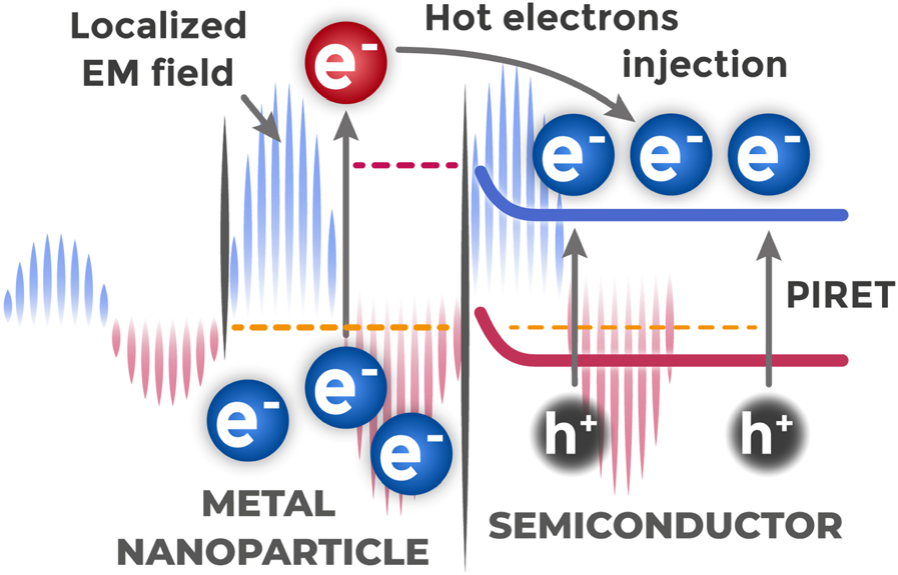
Main mechanisms of plasmonic enhancement in “metal–semiconductor” systems: local field enhancement, “hot” electron injection, and resonant energy transfer.

Absorbed LSPR energy can also decay nonradiatively through Landau damping – a quantum-mechanical effect where collective plasmonic excitation transfers energy to individual metal electrons.^116^ This process generates energetic “hot” electrons with kinetic energy substantially exceeding thermal energy, which enables their injection into the semiconductor conduction band. For this charge carrier injection to contribute to the photoelectrochemical process, these “hot” electrons must possess sufficient energy to overcome the interfacial energy barrier formed at the metal–semiconductor junction. Main plasmonic enhancement mechanisms are illustrated in Fig. 18.

As result of such direct energy transfer from plasmon, individual electrons acquire significant kinetic energy substantially exceeding thermal energy and transition to levels far above Fermi level, becoming “hot” electrons.^130^ For participation in photoelectrochemical process, “hot” electrons must be injected from metal into conduction band of neighboring semiconductor. This transfer is complicated by presence of energy barrier at interface of two dissimilar materials, caused by difference in their electronic properties. “Hot” electrons must have energy exceeding the interfacial barrier height to enter the semiconductor and contribute to the photoelectrochemical response.

In works on creating sensors based on TiO_2_, which itself does not absorb visible light, gold nanoparticles acted as photosensitizers.^131,132^ Under visible light action, they absorbed photons through LSPR, generated “hot” electrons and injected them into TiO_2_ conduction band. The efficiency of this plasmonic enhancement is demonstrated by dramatic improvements in charge transfer kinetics across different Au/TiO_2_ architectures. For Au-decorated TiO_2_ nanorods,^132^ charge transfer resistance dropped by two orders of magnitude from 0.835 MΩ for pristine TiO_2_ to 0.006 MΩ for the Au–TiO_2_ composite under illumination. Similarly, in screen-printed Au/TiO_2_ system,^131^ the charge transfer resistance decreased from 11.50 MΩ for bare TiO_2_ to 3.7 kΩ for the Au-modified electrode under light, confirming that plasmonic sensitization provides substantial improvements in electron transfer regardless of the specific TiO_2_ morphology used. Gold nanoparticles were also applied in systems with bismuth vanadate (BiVO_4_),^133^ also probably aiming to achieve plasmonic effect and improve photoelectric efficiency.

The introduction of metallic bismuth nanoparticles into bismuth-based semiconductors has demonstrated plasmonic enhancement through different mechanisms. In the Bi/BiOI system,^94^ surface plasmon resonance (SPR) effects were confirmed through multiple analytical approaches: the composite showed lower photoluminescence intensity, extended carrier lifetime (1.67 ns *versus* 1.26 ns for pure BiOI), and reduced charge transfer resistance. Hot electrons generated by SPR in metallic Bi nanoparticles are injected into the BiOI conduction band, enhancing charge separation and reducing electron–hole recombination.

Similarly, in BiPO_4_/BiOI heterostructures,^134^ the SPR effect of metallic Bi enhanced both light absorption and electron transfer. The optimized composite exhibited improved absorption in the 400–600 nm range, extended carrier lifetime, and significantly reduced charge transfer resistance. The plasmonic enhancement facilitated electron migration from BiOI conduction band through metallic Bi to BiPO_4_ conduction band, where Cr(vi) reduction occurs.

A similar approach was implemented in the Bi/Bi_2_S_3_/BiVO_4_ system,^135^ where metallic bismuth was introduced to improve photoelectrochemical properties, likely achieving plasmonic enhancement comparable to the other bismuth-based systems.

### 3.4. Conductive networks and charge transport optimization

Photoelectrochemical sensor efficiency is determined not only by quantum yield of charge generation and separation processes, but also by the rate of their subsequent transport from generation site to current-collecting electrode. High analytical signal can be achieved only when photogenerated carriers are transported rapidly and with minimal losses. Charge transfer rate at macroscopic level is limited by overall electrical resistance of the photoelectrode system. Reducing this resistance is one of the tasks in material design, since it leads to increased measured photocurrent at given potential.

At microscopic level, material resistance is determined by two interrelated factors: charge carrier mobility and their lifetime before recombination. Carrier mobility (*μ*) is an intrinsic material characteristic describing how rapidly they can drift under electric field action.^39^ However, even with high mobility, effective transport is possible only if charge carrier manages to reach collector before being annihilated in recombination process. Thus, carrier collection efficiency is determined by kinetic competition between their transport to electrode and recombination processes. Creating branched transport network with high conductivity in composite material bulk is important strategy.^136,137^ Such network forms continuous pathways with low resistance for directed electron movement to current collector, reducing their residence time in material bulk and, consequently, minimizing probability of recombination losses.

The “semiconductor–metal” interfacial boundary controls charge transfer. This boundary forms because contacting materials have different work functions (*Φ*) – the minimum energy required to extract an electron from solid into vacuum. Typically, noble metals used as electrodes have high work function values (for gold 5.31–5.47 eV, for platinum 5.65 eV (ref. 138)). When such materials are brought into contact to align their Fermi levels, electron transfer occurs from semiconductor to metal. This transfer leaves uncompensated positive charge of ionized donors in semiconductor near-surface region, leading to upward energy band bending and potential barrier formation – Schottky barrier.^139^ The field arising in it is completely analogous in its charge separation function to field in p–n heterojunction region.

**Fig. 19:**
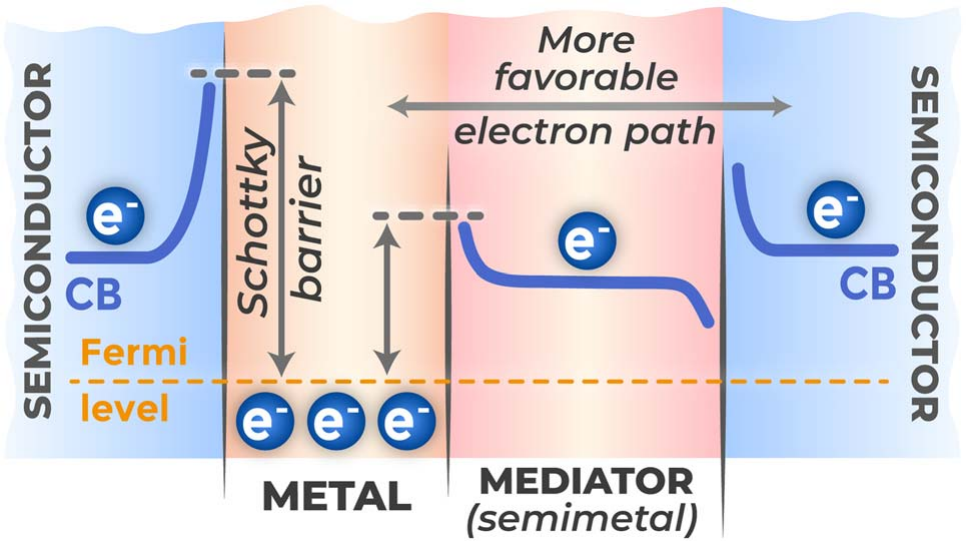
Schematic presentation of Schottky barrier at semiconductor/metal interface and principle of its reduction by mediator introduction.

However, at back contact, whose function is to serve as low-resistance sink for electrons, presence of such barrier has parasitic effect, hindering carrier collection and increasing overall system resistance. An example of solving this problem is contact engineering by introducing graphene interlayer between semiconductor and metal, as implemented in works.^140,141^ Graphene with intermediate work function value replaces one high energy barrier at TiO_2_/Au interface with two sequential and lower ones (TiO_2_/graphene and graphene/Au), as illustrated in Fig. 19. This approach has been demonstrated with substantial performance improvements. In a flexible sensor system,^140^ layer-by-layer graphene deposition enhanced photocurrent more than three times by mediating the Schottky barrier between Au electrodes and TiO_2_. Similarly, another graphene-modified system^141^ achieved approximately 12-fold enhancement in photocurrent intensity while also improving signal stability through more efficient electron–hole pair separation. Schottky barrier nature also explains efficiency of “hot” electron injection from plasmonic nanoparticles into semiconductor, since electron energy must exceed this barrier height.

On the other hand, interface ability to separate charges can be used purposefully at active interface. This same charge separation ability is used when creating hybrid systems with materials possessing high intrinsic electrical conductivity. Such materials form heterojunction with semiconductor that separates charges and simultaneously serves as highly conductive channel for immediate withdrawal of one carrier type. This synergistic effect is realized in MXene/SnS_2_ system,^17^ where Schottky heterojunction provides charge separation and metal-like MXene phase provides their rapid transport. MXenes combine metallic conductivity with hydrophilic surface chemistry.^142^ However, their susceptibility to oxidation in ambient conditions poses long-term stability concerns that must be addressed.^143^

The problem of electron transport lies not only in overcoming final barrier at contact, but also in providing transfer pathway through composite material bulk. To achieve high photocurrent, low ohmic component of entire photoactive layer is necessary. This requires formation of continuous network from conducting components, which should be distributed sufficiently uniformly to service entire photoactive volume, but not in excessive concentration to avoid reducing overall photoactive material content and blocking light access.

**Fig. 20:**
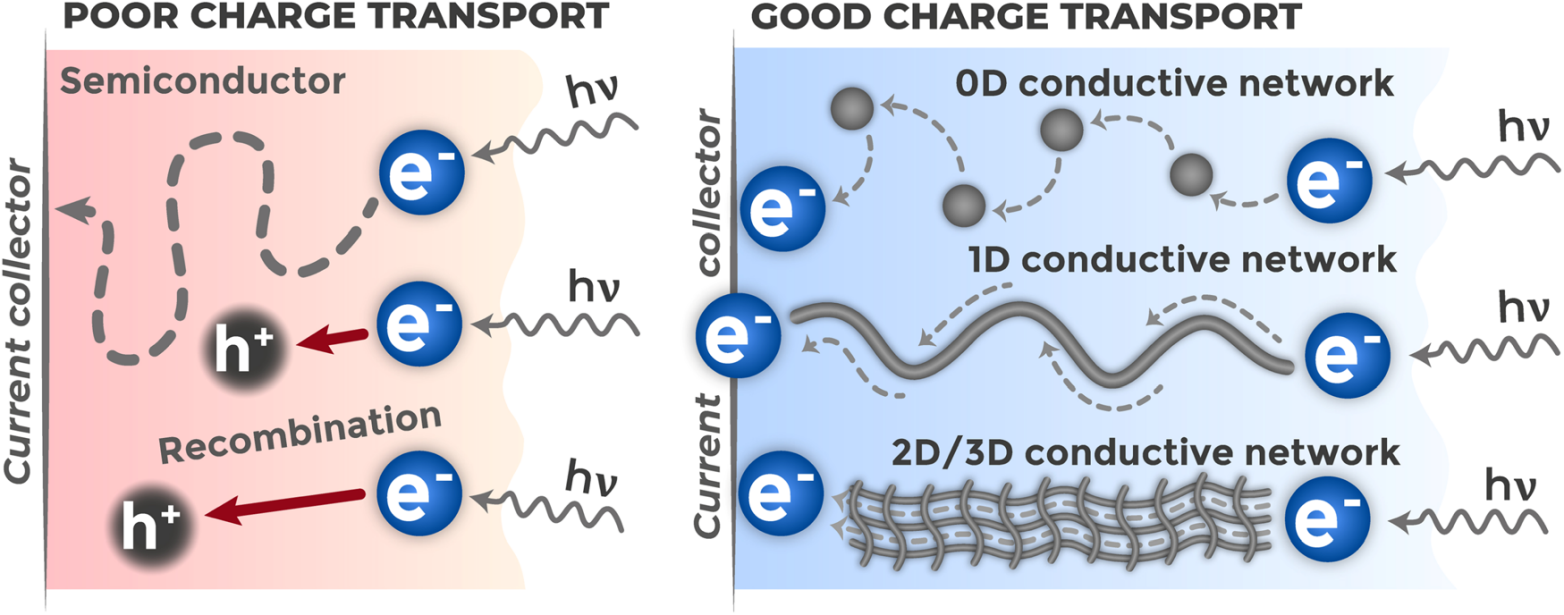
Comparison of inefficient charge transport and improved transport when creating conducting network from 0D, 1D, or 2D/3D materials.

In practice, such transport networks are created by introducing nanocomponents with high conductivity into semiconductor matrix. In AgBr_0.8_I_0.2_–Ag–CNTs composite, this function is performed by one-dimensional carbon nanotubes (CNTs) forming extended conducting channels.^144^ In BiOI–Bi/N-C system, branched network is created simultaneously by two-dimensional sheets of nitrogen-doped carbon and zero-dimensional metallic bismuth nanoparticles.^145^ Zero-dimensional graphene quantum dots (GQD) in TiO_2_/PCV/GQDs system act as discrete centers facilitating charge transfer through synergistic effects. The co-sensitized photoelectrodes demonstrated photocurrents 5 times higher than TiO_2_/PCV and 60 times higher than TiO_2_/GQDs alone, with reduced charge transfer resistance confirming enhanced electron transport.^146^Fig. 20 contrasts poor charge transport in unmodified semiconductors with improved transport achieved through conductive network incorporation.

Alternative approach is using conducting polymers, as in PDAn/CeO_2_/In_2_S_3_ material, where polydopamine (PDA) layer creates continuous conducting framework on semiconductor surface.^147^

### 3.5. Molecular recognition and hybrid sensing platforms

The selectivity of a photoelectrochemical sensor in its basic configuration is determined exclusively by the redox ability of its surface toward solution components. From this perspective, hexavalent chromium, being a strong oxidizer with high redox potential, is a thermodynamically favorable target for reduction by photogenerated electrons. Theoretically, this should provide a certain level of selectivity, since natural waters do not contain many other compounds with comparable oxidizing power. However, this same principle means that any other oxidizer in the sample capable of accepting electrons from the semiconductor will create interfering signal. Besides this, reductants present in the sample may also react with photogenerated holes, changing background photocurrent and also distorting the analytical signal. Thus, selectivity based exclusively on redox potentials is a fundamental problem for simple PEC sensors. To solve it, approaches based on more specific molecular interactions are being developed.

One such approach is indirect detection. Its essence consists in generating analytical response not through direct analyte reaction at the electrode, but through its interaction with a mediator molecule. The main advantage of this method lies in the possibility to use highly specific chemical reaction between mediator and analyte as an additional, non-electrochemical, selectivity filter. It also helps prevent electrode passivation by reaction products, since main interaction occurs in solution.

Practical implementation of this principle is demonstrated in work^88^ using TiO_2_ based sensor, where quercetin is used as mediator molecule. Quercetin in its excited state acts as an electron donor with HOMO–LUMO energy levels suitable for visible light absorption. The energy positions allow electron injection from quercetin's LUMO into the TiO_2_ conduction band, providing photosensitization that enhances the anodic photocurrent approximately 7-fold compared to pure TiO_2_. This electron transfer creates the baseline anodic photocurrent. When Cr(vi) is present in the sample, it oxidizes quercetin, reducing the number of electrons available for transfer to the conduction band and causing photocurrent attenuation. The work demonstrates high method selectivity, particularly in the presence of 500-fold excess of Cr(iii) ions. It should, however, be noted that Cr(iii), being in its highest stable oxidation state, is not an oxidizer for quercetin, so its main possible influence could consist only in non-specific adsorption on electrode surface, not directly affecting the detection mechanism.

Besides the indirect method, work^148^ shows the possibility of increasing selectivity by creating artificial receptors through molecular imprinting. This approach consists in synthesizing polymeric layer on photoelectrode surface in the presence of template ions (Cr(vi)). After their removal, three-dimensional cavities remain in polymer that are complementary to target ions in size and functional group arrangement. In IIP@F-g-C_3_N_4_ system, such ion-imprinted polymer (IIP) provides selective capture of Cr(vi) ions from solution and their delivery to photoactive surface, where their reduction occurs, generating analytical signal. This strategy of using molecularly imprinted polymers to create highly selective recognition sites is not unique to PEC sensors and is also applied in related analytical fields.^149,150^

An approach similar to molecular imprinting in its goal – creating specific recognition centers on surface – consists in forming organo-inorganic hybrid systems. Selectivity in this case is provided by intrinsic chemical properties of pre-selected receptor molecule. Such principle was realized in system based on β-Bi_2_O_3_–Bi_2_WO_6_, whose surface was modified with organic porphyrin – *meso*-tetraphenylporphyrin (H_2_TPP).^151^ In this composite, porphyrin acts as selective receptor, adsorbing Cr(vi) ions from solution through electrostatic interaction with its pyridine and pyrrole nitrogen atoms. This “adsorption–reduction” mechanism, confirmed by theoretical calculations, delivers analyte to the photoactive surface and accounts for the sensor's high selectivity.

Another approach consists not only in surface modification, but also in creating entire photoactive material from pre-designed molecular building blocks. This allows incorporating necessary catalytic and optical properties directly into material bulk. Compounds based on phosphomolybdates, being a subclass of polyoxometalates (POMs), often exhibit photoactivity.^152,153^ This property is due to presence of ligand-to-metal charge transfer transitions (LMCT), when photon excites electron from oxygen atoms (ligand) to vacant d-orbitals of molybdenum atoms, thus creating electron–hole pair. Since these complex crystalline compounds are essentially single-phase semiconductors, they can be used as photoactive materials for direct PEC detection. This possibility is realized in work,^154^ where hybrid material based on Co{P_4_Mo_6_}_2_ clusters and one-dimensional {Co-bimb} chains is used as classical PEC sensor.

However, for many hybrid materials, despite their ability to generate charge carriers, ensuring effective transport of these carriers through complex crystal lattice to form macroscopic photocurrent is non-trivial task. Transport in such systems is often limited by slow hopping mechanism between molecular nodes (clusters).^155^ If rate of these processes is comparable to or lower than recombination rate, most photogenerated carriers annihilate without reaching electrode. To overcome this transport limitation, works^156–158^ apply photo-assisted electrochemistry (PAEC) method.

The PAEC principle consists in using light not as direct signal source, but as catalytic factor for enhancing standard electrochemical measurement. Photogenerated electrons appearing locally on surface are immediately involved in already running, forcibly initiated by external potential, electrochemical process. Using electrochemical methods such as differential pulse voltammetry in PAEC provides additional advantage in form of increased selectivity, since current peak is registered at potential characteristic for specific analyte Cr(vi) reduction. Unlike classical PEC analysis, PAEC method requires more advanced electrochemical techniques such as differential pulse voltammetry and sophisticated data processing.

The PAEC principle consists in using light not as direct signal source, but as catalytic factor for enhancing standard electrochemical measurement. Photogenerated electrons appearing locally on surface are immediately involved in already running, forcibly initiated by external potential, electrochemical process. The effectiveness of this approach is demonstrated through substantial improvements in analytical performance across different polyoxometalate systems. For the hourglass-type phosphomolybdate system,^156^ light illumination increased sensitivity from 216.4 μA μM^−1^ (in darkness) to 330.5 μA μM^−1^ under light, while improving the detection limit from 2.37 nM to 0.95 nM. The wheel-shaped cobalt-phosphate system^157^ showed sensitivity enhancement from 115.60 μA μM^−1^ to 216.76 μA μM^−1^, with detection limit improvement from 46.17 nM to 24.62 nM. Similarly, the hexa-nuclear cadmium cluster system^158^ demonstrated enhanced performance under visible-light assistance, with sensitivity increasing from 106.95 μA μM^−1^ to 226.32 μA μM^−1^ and detection limit improving from 6.25 nM to 4.17 nM.

Using electrochemical methods such as differential pulse voltammetry (DPV) in PAEC provides additional advantage in form of increased selectivity, since current peak is registered at potential characteristic for specific analyte Cr(vi) reduction. Unlike classical PEC analysis, PAEC method requires more advanced electrochemical techniques such as differential pulse voltammetry and sophisticated data processing.

## 4. Critical analysis and methodological challenges

**Fig. 21:**
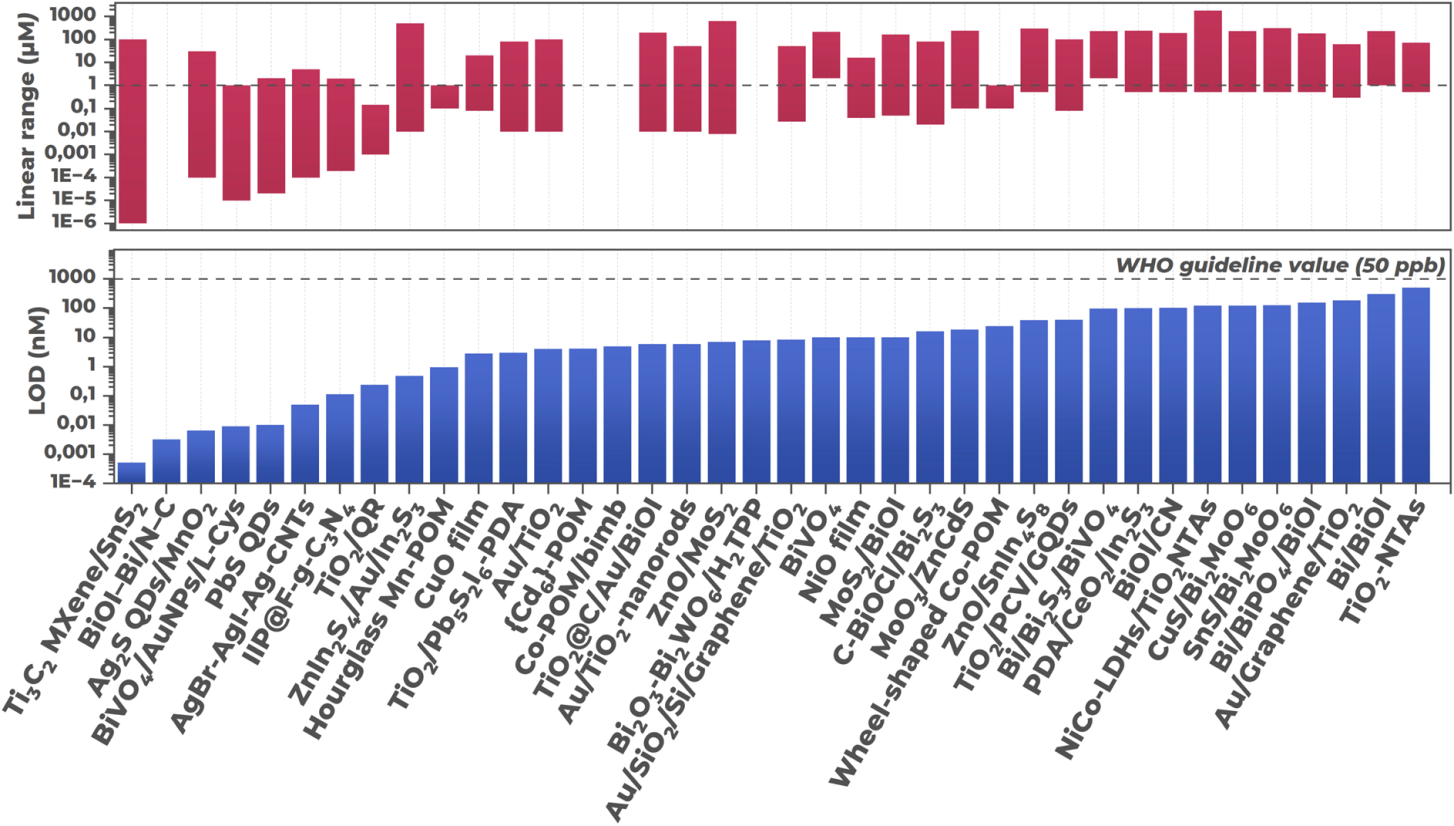
Summary diagram of analytical characteristics of considered PEC sensors for Cr(vi): detection limit (LOD) and linear dynamic range (linear range) compared to WHO standard (0.96 μM). Materials in the diagram: BiVO_4_,^91^ TiO_2_-NTAs,^84^ NiCoLDHs/TiO_2_-NTAs,^105^ CuO film,^87^ NiO film,^95^ CuS/Bi_2_MoO_6_,^82^ TiO_2_/Pb_5_S_2_I_6_–PDA,^85^ MoS_2_/BiOI,^83^ SnS/Bi_2_MoO_6_,^106^ C–BiOCl/Bi_2_S_3_,^107^ MoO_3_/ZnCdS,^108^ TiO_2_@C/Au/BiOI,^92^ ZnIn_2_S_4_/Au/In_2_S_3_,^112^ ZnO/SnIn_4_S_8_,^113^ ZnO/MoS_2_,^114^ Ag_2_S QDs/MnO_2_,^121^ PbS QDs,^86^ Au/TiO_2_,^131^ Au/TiO_2_-nanorods,^132^ BiVO_4_/AuNPs/l-Cys,^133^ Bi/BiOI,^94^ Bi/BiPO_4_/BiOI,^134^ Bi/Bi_2_S_3_/BiVO_4_,^135^ Au/graphene/TiO_2_,^140^ Au/SiO_2_/Si/graphene/TiO_2_,^141^ Ti_3_C_2_ MXene/SnS_2_,^17^ AgBr–AgI–Ag–CNTs,^144^ BiOI–Bi/N-C,^145^ TiO_2_/PCV/GQDs,^146^ PDA/CeO_2_/In_2_S_3_,^147^ BiOI/CN,^93^ TiO_2_/QR,^88^ IIP@F-g-C_3_N_4_,^148^ Bi_2_O_3_–Bi_2_WO_6_/H_2_TPP,^151^ Co-POM/bimb,^154^ hourglass Mn-POM,^156^ wheel-shaped Co-POM,^157^ {Cd_6_}-POM.^158^

Application of the materials strategies discussed in the previous section aims primarily at achieving detection limits lower than the maximum allowable concentration established by the World Health Organization (WHO) for hexavalent chromium in drinking water (0.96 μM).^134^ As shown in Fig. 21, all PEC sensors reviewed in this work demonstrate LOD values significantly below this threshold, confirming their potential suitability for practical water quality monitoring applications. Most systems successfully cover several orders of concentration magnitude in their linear ranges.

Among the reviewed systems, several achieve exceptionally low detection limits. A composite based on Ti_3_C_2_ MXene/SnS_2_ (ref. 17) achieved 0.51 pM with a linear range spanning from 1.0 pM to 0.1 mM. The performance likely stems from synergy at the material interface: a Schottky heterojunction separates charges while the metallic MXene phase provides rapid electron transport, suppressing recombination. The BiOI–Bi/N-C composite^145^ (LOD 3.2 pM) similarly employs a network of N-doped carbon sheets and metallic bismuth nanoparticles to facilitate charge transport. The AgBr–AgI–Ag–CNTs sensor^144^ (LOD 50 pM) combines carbon nanotube conductive pathways with metallic silver nanoparticles, which potentially contribute through plasmonic enhancement, alongside charge-separating effects at multiple internal heterojunctions.

Semiconductor quantum dots provide another pathway to low detection limits. Sensors using PbS QDs^86^ and Ag_2_S QDs/MnO_2_ (ref. 121) reached 10 pM and 6.46 pM, respectively. The narrow bandgaps enable visible light absorption, and performance may benefit from multiple exciton generation.

The ZnIn_2_S_4_/Au/In_2_S_3_ Z-scheme heterostructure^112^ attained 0.48 nM detection limit. Among surface-modified systems, the ion-imprinted polymer sensor IIP@F-g-C_3_N_4_ (ref. 148) reached 115 pM through selective Cr(vi) recognition sites. The BiVO_4_/AuNPs/l-Cys system^133^ (LOD 9.1 pM) employed an l-cysteine self-assembled monolayer to reduce background current, improving signal-to-noise ratio. Indirect detection strategies also demonstrate exceptional sensitivity, as exemplified by the TiO_2_/quercetin sensor^88^ (LOD 0.24 nM). However, beyond sensor design itself, the measurement environment substantially influences actual analytical performance. These characteristics vary with experimental conditions, particularly solution acidity.

Measurements in most works are conducted under strictly controlled conditions, most often in acidic or near-neutral media, and buffer solutions such as PBS,^84,88,146^ Tris–HCl buffers,^85,87,148^ as well as dilute solutions of strong acids such as HNO_3_ (ref. 82, 94 and 134) and HCl^131,132^ are used to maintain constant pH. Medium selection is not arbitrary, since acidity simultaneously determines the predominant ionic form of Cr(vi), its redox potential, semiconductor energy band position, and surface reaction kinetics.

Depending on acidity and total chromium concentration in solution, equilibrium is established between its various forms: chromic acid (H_2_CrO_4_), hydrochromate ion (HCrO_4_^−^), chromate ion (CrO_4_^2−^), and dichromate ion (Cr_2_O_7_^2−^), according to equations:

5 H_2_CrO_4_ = HCrO_4_^−^ + H^+^

6 HCrO_4_^−^ = CrO_4_^2−^ + H^+^

7 2HCrO_4_^−^ = Cr_2_O_7_^2−^ + H_2_O

**Fig. 22:**
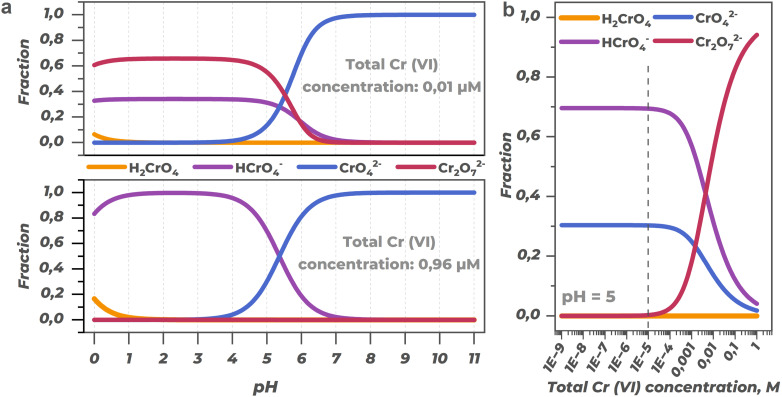
Distribution diagrams of Cr(vi) ionic forms in aqueous solution depending on pH (a) and total chromium concentration (b). Diagrams calculated based on eqn (4)–(6), using dissociation constants: lg *K* = 0.7 (reaction (5)),^159^ lg *K* = 5.36 (reaction (6)),^160^ lg *K* = 2.45 (reaction (7)).^160^

Distribution of main Cr(vi) forms is shown in Fig. 22 diagrams depending on pH and total chromium concentration. As seen from Fig. 22a, regardless of concentration, chromate ion (CrO_4_^2−^) predominates in neutral and alkaline regions. At the same time, in acidic medium, form distribution changes substantially with concentration change: at high concentrations dichromate ion (Cr_2_O_7_^2−^) dominates, while at low concentrations – hydrochromate ion (HCrO_4_^−^). Chromic acid (H_2_CrO_4_) concentration becomes significant only in strongly acidic pH region. The graph in Fig. 22b demonstrates that in a certain pH range (distribution at pH = 5 shown as example), form distribution remains relatively stable in a wide region of low concentrations.

Acidity control during analysis is needed for consistent results. Moreover, pH directly influences thermodynamic characteristics of the detection process. All reduction reactions of Cr(vi) forms to Cr(iii) are proton-dependent processes, so their redox potential varies strongly with medium acidity.

**Fig. 23:**
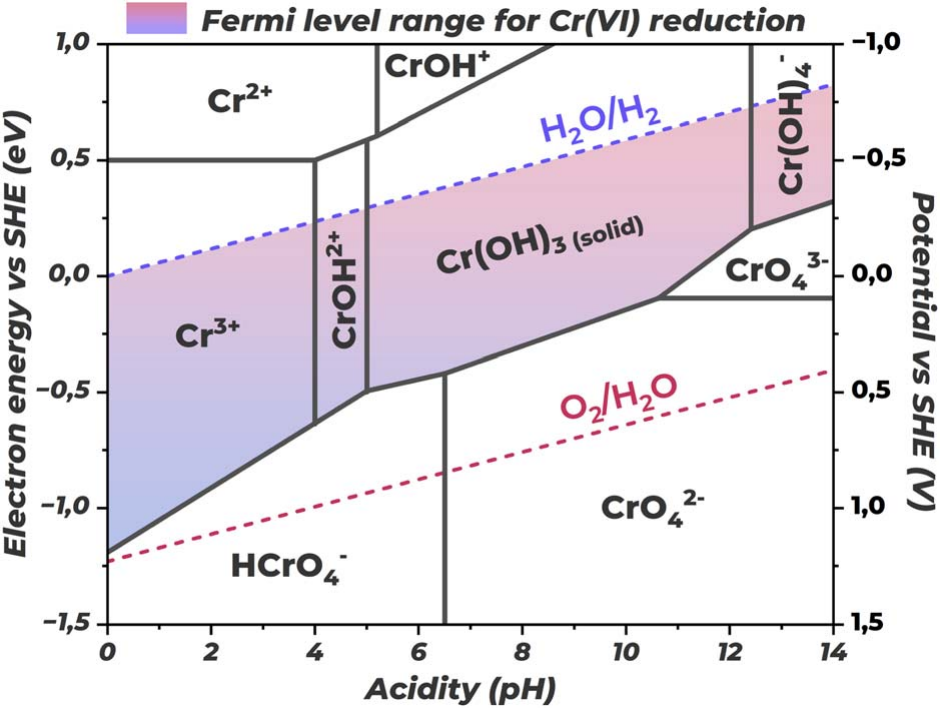
Modified Pourbaix diagram (potential scale inverted), adapted from ref. 161. Published by Taiwan Association for Aerosol Research under the CC BY 4.0 license.

The Pourbaix diagram for chromium species (Fig. 23) illustrates these pH-dependent relationships, where the left axis presents negative potential values corresponding to electron energy and the right axis shows traditional electrochemical potentials. As seen from the diagram, the redox potentials of all Cr(vi) forms increase with decreasing pH (increasing acidity), making them more thermodynamically favorable for reduction in acidic conditions.

This explains why many sensors, despite unstable chromium form distribution in acidic media depending on concentration, use acidified buffer solutions or acids. Thermodynamic driving force for reduction, changing at different solution concentrations, will directly influence electron transfer process from sensor to chromium. Simultaneously, the semiconductor surface state itself changes. For many oxide materials, flat band potential (*U*_fb_) depends linearly on pH due to protonation or deprotonation of surface hydroxyl groups.^162,163^ This leads to energy band bending change at fixed external potential, affecting photogenerated charge separation efficiency. Thus, pH change simultaneously modifies both thermodynamic driving force for analyte reaction and electronic structure of photoelectrode itself.

pH can also determine the detection mechanism itself. The signal-weakening mechanism based on electrode surface blocking by insoluble Cr(OH)_3_ hydroxide works only in neutral or weakly alkaline media. For sensors based on CuO and NiO films,^87,95^ measurements are conducted in neutral buffer solutions because in acidic media the formed Cr(iii) will not precipitate on the surface and cause steric blocking.

The analytical signal dependence on pH can be complex and show extrema. For the Ti/TiO_2_–Pb_5_S_2_I_6_–PDA sensor,^85^ maximum photocurrent was achieved at pH 5.0. At higher pH values (>5.5), the signal decreased due to Cr(OH)_3_ precipitation and catalyst deactivation. At lower pH, it decreased due to competing capture of photogenerated electrons by protons. Optimal pH is a compromise that provides both high Cr(vi) oxidizing ability and prevents side processes. The shaded region in the Pourbaix diagram represents the thermodynamic window where the semiconductor Fermi level enables Cr(vi) reduction to Cr(iii) without concurrent water reduction to hydrogen.

The position of the Fermi level relative to these redox energies determines both the thermodynamic feasibility and kinetic efficiency of electron transfer. When the Fermi level lies at higher energy than the Cr(vi) reduction level (more negative potential than Cr(vi)/Cr(iii)), electron transfer becomes favorable, with the driving force increasing as the energy difference grows. However, if the Fermi level reaches too high energies and approaches the water reduction level (more negative H_2_O/H_2_ potential), competing water reduction may occur. The optimal zone is bounded by the Cr(vi) reduction levels at lower energies and the water reduction level at higher energies.

One may notice that Cr(OH)_3_ precipitation becomes thermodynamically favorable starting from pH 4.5, yet many sensors operating in neutral media demonstrate signal-on mechanisms instead of the expected signal-weakening from surface blocking. This can be explained by kinetic competition between processes. The signal-on response occurs through rapid electron consumption by Cr(vi), immediately enhancing photocurrent, while Cr(OH)_3_ precipitation is a slower secondary process. Additionally, chromium reduction involves charge sign change from anionic Cr(vi) forms to cationic Cr(iii), and the formed Cr^3+^ cations experience electrostatic repulsion from the positively biased photoanode surface, promoting precipitation in solution rather than surface blocking.

Photocurrent behavior varies depending on whether sensors employ signal-on or signal-off mechanisms. Most sensors demonstrate “signal-on” mechanism, where response grows from low background levels. This approach is considered more reliable since signal appears only in response to analyte, reducing the probability of false-positive results and often providing wider dynamic range. “Signal-off” mechanism, where a high stable signal decreases in analyte presence, can achieve very high sensitivity at ultra-low concentrations, since detecting small changes in strong signals is easier than detecting weak signal appearance. However, such systems may be prone to false alarms – any factor reducing signal (impurity adsorption, electrode degradation) can be mistakenly interpreted as analyte presence. This is particularly problematic in real samples containing organic matter, suspended particles, or other electroactive species.

**Fig. 24:**
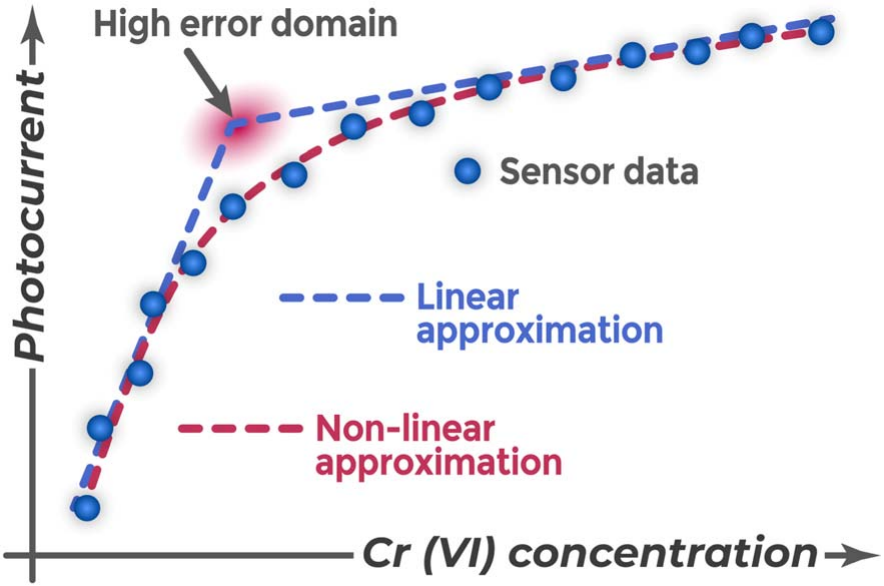
Comparison of linear and nonlinear approximation of sensor calibration curve.

In ideal case, sensor response can be linear in certain concentration range, as shown for several systems.^85,86,131,132^ However, linear dependence is rare in wide concentration range. Often nonlinear response is observed, for which researchers use two main approaches. Most common is piecewise-linear approximation, where the nonlinear curve is divided into sections, each described by a linear equation.^105,134^ More rigorous is using a single nonlinear equation to describe the entire range – often logarithmic dependence.^91,140,141^ As shown in Fig. 24, piecewise-linear approximation can lead to significant errors in concentration determination, especially in transition regions between sections, while single nonlinear equations provide more accurate description throughout the working range. However, nonlinear approach requires more complex mathematical data processing, sensitivity becomes variable quantity across concentration range, complicating comparison of different sensor systems. Moreover, nonlinear models may give less reliable results when extrapolating beyond calibration range.

Advantage of photoelectrochemical method over total elemental analysis methods (*e.g.*, AAS^164^) is its intrinsic, inherent to mechanism itself, selectivity toward different chromium oxidation states. Sensors respond to electrochemically active Cr(vi) form, while Cr(iii), inert under given conditions, does not participate in redox processes on photoelectrode surface and does not create direct interfering signal. In addition, many works demonstrate high selectivity of developed sensors toward wide spectrum of other potentially interfering inorganic cations and anions, such as Na^+^, K^+^, Ca^2+^, Mg^2+^, Cu^2+^, Fe^3+^, Cl^−^, NO_3_^−^, and SO_4_^2−^, even at their significant excesses.^84,85,87,91,146^

Nevertheless, as discussed in Section 3.5, fundamental detection mechanism based on redox reaction is inherently non-specific toward other electroactive substances in sample. Many studies report the ability to detect various electroactive analytes beyond Cr(vi),^135,154^ which undoubtedly is an advantage for multi-analyte screening but simultaneously proves fundamental questions about sensor selectivity in real samples. Due to this, best test of practical applicability and real selectivity of sensor is its testing on samples from real objects. Many researchers successfully applied their developments for analysis of tap,^91^ river,^105^ lake,^134^ and waste water,^85^ as well as food product samples.^112^ For evaluating measurement accuracy in complex matrix, spike-and-recovery test is often used, where known amount of Cr(vi) is added to sample and what fraction was successfully detected by sensor is determined. For most considered systems, this indicator is in acceptable range of 90–103%.^82,83,91,93,131^ Nevertheless, to achieve true specificity in complex and diverse composition samples, most promising is not so much improving semiconductor properties itself, but its modification with specific recognition elements, which was considered in Section 3.5.

Besides selectivity, other operational characteristics such as stability, reproducibility, and response time are important for practical sensor application. Most developed systems show good performance for these parameters. Sensor stability is often evaluated both short-term (photocurrent stability under continuous or intermittent illumination) and long-term (characteristic preservation during storage). Many systems retain more than 90–95% of their initial activity after several weeks and even months of storage, and withstand hundreds of measurement cycles with minimal signal degradation.^82–84,91,131^ Reproducibility of different sensors is also at high level: relative standard deviation (RSD) for measurements on different sensors usually does not exceed 5%.^82,83,85,91,131^

As shown in our previous work,^5^ hexavalent chromium contamination problem is large-scale and can cover large territories, requiring mass screening. In this context, such parameter as portability becomes important. In ideal case, PEC sensor could consist only of working photoelectrode, counter electrode, light source, and simple ammeter measuring flowing current. However, in practice, considered Cr(vi) sensors operate in potentiostatic mode in three-electrode cell, with applied external, controlled potential, which requires potentiostat presence.

“Unbiased” sensors operating at 0 V potential relative to reference electrode are often mentioned in works.^83,87,105,140,141^ However, this mode does not mean absence of external control. In this case, potentiostat actively works, supplying necessary voltage between working and auxiliary electrodes to forcibly maintain zero potential difference between working electrode and reference electrode. This is not the same as truly self-sufficient (galvanic) two-electrode system where current flows spontaneously.

Controlled potential requirement stems from fundamental thermodynamic limitation. For system to work spontaneously in galvanic mode, total electromotive force (EMF) of cell, equal to potential difference at cathode and anode, must be positive. In case of photocathodic Cr(vi) reduction, conjugated reaction at counter electrode (often made of inert platinum) should be oxidation of solution component. In aqueous media, such reaction is water oxidation itself, which has very high thermodynamic potential (+1.23 V *vs.* SHE)^77^ and, moreover, is kinetically hindered process.

Therefore, to ensure current flow in the circuit, instead of inert materials, photoactive counter electrodes should be considered that can provide the conjugate reaction under light illumination. As demonstrated earlier in this review, Cr(vi) sensors operate in both photocathodic and photoanodic modes depending on the semiconductor type and applied potential. This versatility enables the use of two different sensor types that both detect chromium, operating either through the same mechanism (both signal-on or both signal-off) or complementary mechanisms. Such dual-photoelectrode configurations require careful engineering to ensure optimal thermodynamic matching and synchronized operation.

This approach has been demonstrated in practice, such as in self-powered sensors integrating both photocathode and photoanode configurations for microplastic detection.^165^ These systems achieve autonomous operation without external power sources while maintaining analytical accuracy.

Nevertheless, the potentiostat requirement may not be as critical as it appears, since portable and inexpensive potentiostats are actively being developed, including Arduino-based platforms.^166^ Recent research has produced highly precise, versatile measurement systems incorporating three-electrode potentiostats with integrated LED excitation sources, offering wireless smartphone control and multiple wavelength capabilities.^167^ These developments make potentiostatic control increasingly accessible for field applications.

## 5. Summary and prospects

Research in photoelectrochemical sensors for hexavalent chromium detection spans from fundamental semiconductor physics principles to sophisticated multi-component analytical platforms. The field encompasses both simple single-phase semiconductor systems and complex heterostructural architectures. Single-phase systems, while straightforward in design, often suffer from rapid charge recombination. Heterostructures address this limitation through type-II band alignment and Z-scheme mechanisms that spatially separate charge carriers.

Various materials strategies have been explored to overcome fundamental limitations: heterostructures and Z-schemes for charge separation, quantum dots for enhanced light absorption and multiple exciton generation, plasmonic nanostructures for visible light activation, conductive networks for improved charge transport, and molecular recognition elements for enhanced selectivity. These approaches collectively established foundations for sensitive Cr(vi) detection. The reviewed sensors consistently achieved detection limits well below WHO standards, with many systems reaching nanomolar detection limits and good selectivity against interfering species.

Persistent challenges limit practical deployment. The pH dependence of both chromium speciation and semiconductor band positions complicates measurements in unbuffered environmental samples. Most current systems require potentiostatic control, which adds complexity to portable operation. While analytical performance has been well-established, engineering practical field-deployable systems remains the primary challenge.

Future research should concentrate on developing integrated monitoring platforms rather than improving individual sensor components. The wealth of existing photoelectrochemical systems provides sufficient analytical capabilities; the field now requires portable instrumentation and continuous online monitoring capabilities. Dual-photoelectrode configurations offer promise for autonomous operation, where photocathode and photoanode can operate in galvanic mode without external potential control. The development of monitoring systems incorporating solar panels, energy storage, and wireless data transmission could enable deployment in remote locations where chromium contamination from geogenic sources requires surveillance but power infrastructure is unavailable. Such systems would address monitoring needs for chromium contamination, which affects vast territories through both industrial and natural sources.

Machine learning integration offers pathways for overcoming matrix effects and pH dependencies that currently limit sensor performance in real samples. Algorithms could compensate for environmental variables while maintaining analytical accuracy across diverse sample conditions.

The understanding developed through chromium sensor research transfers directly to other environmental contaminants. The materials strategies and interface engineering methods established here provide frameworks for detecting other heavy metals and organic pollutants.

Practical deployment will require interdisciplinary collaboration between materials scientists, electrochemists, and instrumentation engineers. The transition from laboratory demonstrations to field-ready monitoring systems presents the field's next major development phase, where engineering practicality must be balanced with analytical performance requirements.

## Author contributions

Yaroslav Zhigalenok: conceptualization, methodology, investigation, visualization, writing – original draft, writing – review & editing. Aigerim Tazhibayeva: validation, writing – original draft, writing – review & editing. Saule Kokhmetova: conceptualization, supervision, project administration, funding acquisition, writing – review & editing. Alena Starodubtseva: investigation, data curation, writing – review & editing. Tatyana Kan: investigation, resources, writing – review & editing. Fyodor Malchik: methodology, formal analysis, writing – review & editing.

## Conflicts of interest

There are no conflicts to declare.

## Data Availability

No primary research results, software or code have been included and no new data were generated or analysed as part of this review.
